# Emulgels Containing Propolis and Curcumin: The Effect of Type of Vegetable Oil, Poly(Acrylic Acid) and Bioactive Agent on Physicochemical Stability, Mechanical and Rheological Properties

**DOI:** 10.3390/gels7030120

**Published:** 2021-08-12

**Authors:** Rafaela Said dos Santos, Jéssica Bassi da Silva, Hélen Cássia Rosseto, Camila Felix Vecchi, Katieli da Silva Souza Campanholi, Wilker Caetano, Marcos Luciano Bruschi

**Affiliations:** 1 Postgraduate Program in Pharmaceutical Sciences, Laboratory of Research and Development of Drug Delivery Systems, Department of Pharmacy, State University of Maringa, Maringa 87020-900, PR, Brazil; rafaelasaids@gmail.com (R.S.d.S.); jessicabassidasilva@gmail.com (J.B.d.S.); helenrosseto@gmail.com (H.C.R.); camilaf.vecchi@gmail.com (C.F.V.); 2 Postgraduate Program in Chemistry, Department of Chemistry, Research Nucleus in Photodynamic Systems, State University of Maringa, Maringa 87020-900, PR, Brazil; katieli_souza@hotmail.com (K.d.S.S.C.); wcaetano@uem.br (W.C.)

**Keywords:** gels, emulgels, physicochemical properties, mechanical, rheology, bioadhesion, poly(acrylic acid) derivatives

## Abstract

Emulgels are obtained by the entrapment of an organic phase within a three-dimensional network built by hydrophilic molecules. Polymers based on cross-linked poly(acrylic acid) have been utilized as gel matrices, improving adhesiveness, rheological and mechanical performance. Propolis (PRP) produced by *Apis mellifera* L. bees displays a wide range of biological activities. Together with curcumin (CUR), they may show synergic anti-inflammatory, antioxidant and antimicrobial action on skin disorders. This work investigated the effect of vegetable oils (sweet almond, andiroba, and passion fruit) with regard to the physicochemical properties of emulgels composed of Carbopol 934P^®^, Carbopol 974P^®^, or polycarbophil aiming the CUR and PRP delivery. Physicochemical stability enabled the selection of systems containing passion fruit or andiroba oil. Mechanical and rheological characteristics provided rational comprehension of how vegetable oils and bioactive agents affect the structure of emulsion gels. All formulations exhibited high physiochemical stability and properties dependent on the polymer type, oil, and bioactive agent. Formulations displayed pseudoplastic, thixotropic and viscoelastic properties. Emulgels containing andiroba oil were the most stable systems. Carbopol 934P^®^ or polycarbophil presence resulted in formulations with improved smoothness and mechanical properties. Systems containing andiroba oil and one of these two polymers are promising for further investigations as topical delivery systems of CUR and/or PRP on the skin and mucous membranes.

## 1. Introduction

Wound healing devices represent an important segment of the global medical care market. However, the development of innovative topical dosage forms is extremely challenging in clinical practice, as they require systems capable of being retained on the surface of application for a prolonged time, allowing drug transportation through the stratum corneum (SC). Highly viscous materials are known to resist to the stress and movement (shear rates) found in the skin and mucosal surfaces [1]. Polymer gels drug delivery systems are typically viscous and usually display a pseudoplastic or thixotropic profile to aid the spreading of the dosage form during the application. Hence, many investigations have emerged that address the state of the art of gel-based drug delivery, focusing on the requirements for the promotion of rapid and successful wound healing.

Gels can be cross-linked polymeric systems able to retain solvent and particles, providing controlled drug release. Depending on their composition, they can also display bioadhesive properties, which can improve the residence time at the site of administration site. For instance, poly(acrylic acid) derivatives are reported as suitable bioadhesive polymers, offering improved adhesiveness of pharmaceutical systems to the skin and mucosal surface [2,3,4,5]. Although many kinds of research have been performed in this field, a small number of gels-based drug delivery systems are approved by Food and Drug Administration (FDA) [6]. The optimization of these systems regarding drug loading capacity, mechanical behavior, bioadhesion, and control of drug release kinetics may favor the development of advanced drug delivery systems.

Emulsion-filled gels are matrices of polymeric gels into which oil droplets are incorporated (emulgels). These systems can combine emulsion and gel properties, acting as dual control release systems for hydrophilic and hydrophobic drugs [7]. These composite materials can be obtained by the entrapment of an organic phase (e.g., vegetable oils) within a three-dimensional network built by hydrophilic molecules [8]. The gelling ability of the external phase can immobilize the hydrophobic phase due to the formation of physical or chemical gels, respectively, by entangling or crosslinking of chains [9]. They can display good rheological properties of the gels, being easily spreadable and removable. Moreover, emulsified gels have proven to be a suitable vehicle for poorly water-soluble drugs, as they are also an emollient system with a long shelf life [10,11]. These systems can be administered by different routes and for the delivery of many bioactive agents [9,10,12]. Topical administration on the skin or mucosal surfaces of emulgels is an attractive alternative to conventional oral therapy since they avoid the first-pass metabolism and promote greater patient compliance [13,14,15].

The type and concentration of the polymer composing the gel matrix can influence the stability and the release rate of the incorporated drug [7]. Moreover, bioadhesive polymers based on cross-linked poly(acrylic acid), such as carbomers and polycarbophil, have been extensively used to improve several other bioadhesive, rheological and mechanical performance of semisolid systems [2,16,17,18,19,20,21]. Poly(acrylic acid) derivatives can swell almost 10 times their original size to form a gel when exposed at pH 4.0 to 6.0, reaching maximum drug release at higher pH values [22]. The carboxyl groups in the acrylic acid chains are the main ones responsible for many characteristics of these polymers. Differences among poly(acrylic acid) derivatives are mainly related to the cross-linking degree and manufacturing conditions [18,23]. Carbopol 934P^®^ (C934P) and Carbopol 974P^®^ (C974P) are high molecular weight polymers cross-linked with allyl sucrose and allyl pentaerythritol, respectively. They display good bioadhesive and viscoelastic properties [23,24]. Polycarbophil (PC), a poly(acrylic acid) derivative cross-linked with divinyl glycol, has been highly used in commercial preparations. When applied to the skin or mucous membrane, the safety and absence of irritating effects make their use allowed by FDA [3].

Whether used in their native form or as emulsion formulations, natural oils are often used by the pharmaceutical industry as a therapeutic enhancer, emollient, or as a vehicle for hydrophobic drugs. Plenty of oils prevent injuries in various skin healing stages, such as andiroba oil and sweet almond oil [25,26]. Some of them may still display bactericidal activity and protective action against damages by solar radiation [27]. Passion fruit oil, for instance, is a highly antioxidant oil used to prevent premature aging [28]. Usually, vegetable oils have low viscosity and molecular weight, making them less occlusive than mineral oils, with suitable penetration into the skin [29,30]. The antioxidant compounds (phenolic compounds) comprising natural oils have been linked to their high stability, as already observed for sweet almond oil [31,32]. The literature reports the use of natural oils as organic solvents over emulgels preparation [10,12]. Andiroba oil, passion fruit oil, and sweet almond oil have been reported in emulgels composed of C934P, for instance [33], with andiroba oil frequently demonstrating high stability of the emulsion systems in comparison to other oils [27].

The use of multiple antioxidant and healing chemotherapeutic agents can favor the treatment of skin lesions. Recent studies have shown the synergistic effects of propolis extract (PRP) and curcumin (CUR) increasing the therapeutic efficiency, mainly due to their antioxidant, anti-inflammatory and antimicrobial activities [34,35]. The PRP is a complex mixture of vegetable substances used to protect the comb by sealing gaps and keeping insects and microorganisms away [36,37]. It displays an extensive range of biological activities, going from antioxidant, immunostimulant, antimicrobial, anti-inflammatory, and antiviral effects to anticancer activity [36,38,39] to tissue regenerating and healing in ulcers [38,40]. According to Abd-Elrazek and collaborators (2020), PRP can inhibit systemic inflammation, as well as decrease the oxidative stress by protecting the membranes and eliminating free radicals, evidencing its anti-inflammatory and antioxidant activities [41]. This drug is frequently processed as ethanolic extract (PE), which can be used as a final or intermediary dosage form [24,38,42]. A satisfactory dispersion of PRP constituents into pharmaceutical formulations is dependent on the physicochemical properties of the systems, and their use in combination with emulgels has already been reported as a suitable strategy for the delivery of PRP chemotherapeutic agents in topical therapy [33].

Extracted from Curcuma longa Linn. [43], curcumin (CUR) is present in curry and saffron, common spices in typical dishes from India and China. CUR presents itself in the structural formula of two meta-phenolic rings substituted with methyl ether, tautomeric ketoenolic groups, and unsaturated carbon chain, contributing to its lipophilicity (log P = 2.5). The aversion of CUR to water allows it to cross the plasma membrane freely and accumulate within the cells of lipid tissues. CUR has three ionization constants (pKa): 8.38; 9.88 and 10.51, which ensures a basic character, with stable behavior at low pH (from 1 to 6) and unstable at the higher ones [44,45,46,47]. This bioactive agent is widely studied for the treatment and prevention of several diseases such as cystic fibrosis, psoriasis, depression, asthma, arthritis, brain injury, diabetes and healing. It displays anti-inflammatory, antioxidant, and antimicrobial action and helps to prevent cardiovascular diseases [44,45,48,49].

Thus, this work demonstrates the design and optimization of emulgels composed of the passion fruit, andiroba and sweet almond emollients therapeutic oils, combined with PRP and CUR in polymeric matrices of C934P, C974P or PCB. A factorial design coupled with the mechanical and rheological properties of the systems led to understanding the important effects of composition on the physicochemical stability of the emulgel, composed of therapeutic oil microvesicles containing solubilized CUR.

## 2. Results and Discussion

### 2.1. Preparation and Microarchitecture of Emulgel Systems

**Figure 1:**
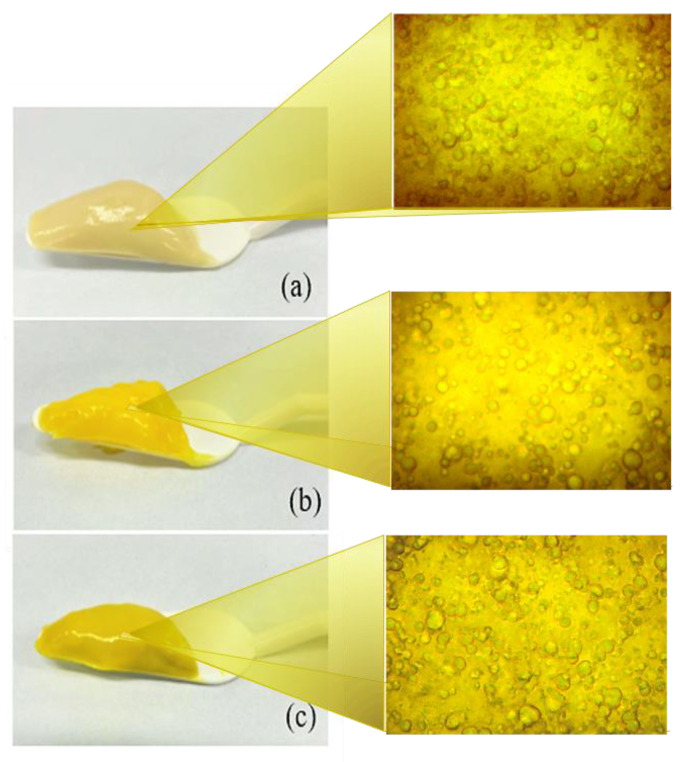
Macroscopic and microscopic (original magnification ×400) characteristics of emulgel systems containing andiroba oil (8%, *w*/*w*), Carbopol 934P^®^ (1%, *w*/*w*), and: (**a**) propolis extract (PE; 8%, *w*/*w*); (**b**) curcumin (CUR; 0.1%, *w*/*w*); (**c**) PE (8%, *w*/*w*) plus CUR (0.1%, *w*/*w*).

The emulgels were prepared using both poly(acid acrylic) derivatives, vegetable oils, and combinations of bioactive agents (PRP and CUR). After pH neutralization of the emulgels, the polymer chains of the gelled continuous phase showed a higher density of the ionized carboxylic groups, fostering electrostatic repulsion and osmotic effects between the internal and external environment, which increases their viscosity [19,23,43,50,51]. As reported in the literature, the addition of oil plays an important role on hardness due to the creaminess acquired by the products [1]. The macroscopic characteristics (e.g., color and overall appearance) of each formulation were dependent on the presence of the bioactive agents (Figure 1).

The milky nature of the systems revealed the presence of droplets on a microscopic scale. The knowledge of the microarchitecture of the emulsions was ascertained by performing optical microscopy, which showed the presence of either dispersed or partially aggregated circular droplets distributed in the aqueous gel phase. The interfaces were well-defined and bounded by the mesh-like channels formed by cross-linking polymeric structuring agents. The physicochemical properties of the gelling polymers and oil droplets in the emulsion (size, distribution, and interactions with the polymer network) determined the stability, mechanical and rheological characteristics of these systems.

The following studies complement the understanding of the microarchitecture of emulsions, showing the influence of thermal, temporal and physicochemical effects on the stability of the systems. Studies will allow the development of a higher quality product as they evaluate the effects of the type of emollient, polymer and drug on the coalescence tendency of emulgels.

### 2.2. Physicochemical Stability Study

The thermodynamics of emulsion formation describes a non-spontaneous process that leads to an increase in Gibbs energy (ΔG= ΔAγ – TΔS). The ΔAγ component (area of the droplets and γ is the interfacial tension) is generally positive and cannot be overcome by the dispersion entropy (TΔS), justifying the thermodynamic instability of emulsified systems (a natural tendency for these systems to coalesce) and its priority investigation [52,53].

**Figure 2:**
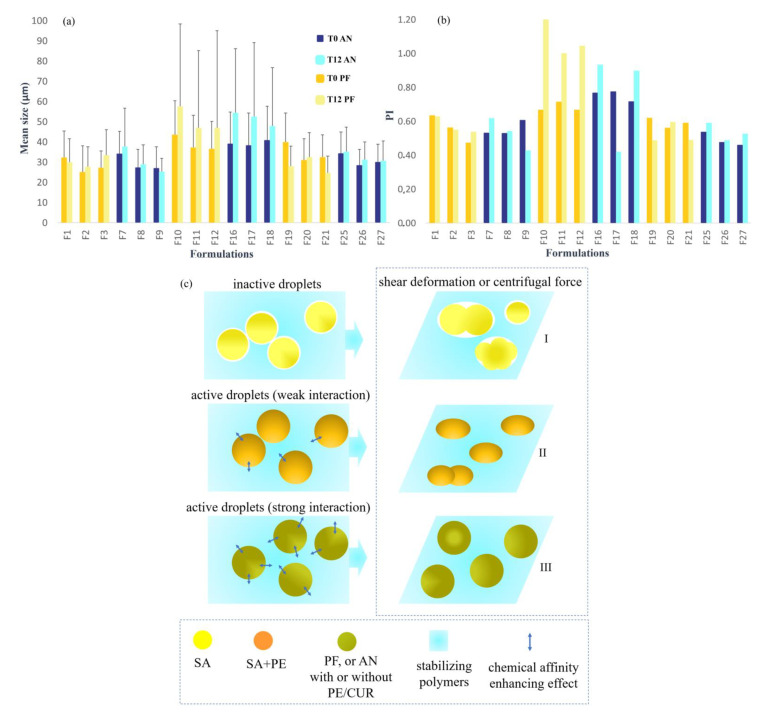
Size analysis of inner phase globules of emulsion systems containing passion fruit (PF) or AN (andiroba) oils just after the preparation (T0) and after 12 days (T12) of the ice-thawing cycles. (**a**) Mean globule size; (**b**) polydispersity index; (**c**) schematic representation of the behavior of different types/compositions of oil droplets relative to the data from size analysis, displaying inactive droplets (I) and active droplets with weak (II) and strong interaction (III).

The mean droplet sizes of the inner phase of formulations and their polydispersity index (PI) were determined at T0 and T12 (Figure 2, Supplementary Table S1).

**Figure 3:**
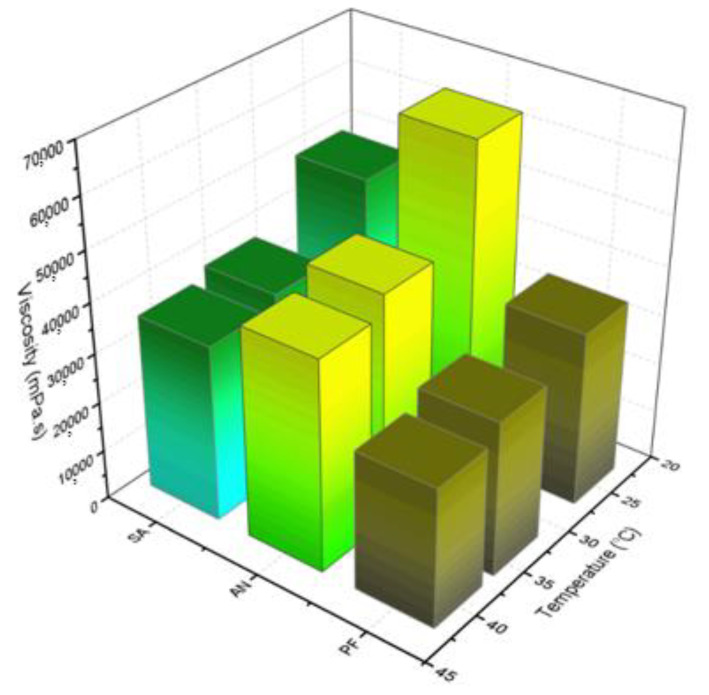
Viscosity (mPa.s) of sweet almond (SA), andiroba (AN) and passion fruit oils at 25, 34 and 40 °C. Each value represents the mean value of four replicates.

The area of the droplets into the emulgel were initially dependent on the energy applied over the manufacturing process (vigorous stirring effect) and the interfacial force promoted by polymer and natural surfactants from the vegetable oils. The milk nature and coloration of the emulsions were preserved even after the six freeze–thaw cycles, suggesting physicochemical stability of the emollients and drugs. The sizes at T0 ranged from 30 µm to 40 µm, and the PI showed satisfactory homogeneity at the initial time (PI close to 0.60, Figure 2a,b). However, the repetitive thermal cycles fostered coalescence effects, consequently increasing the droplet diameter for most systems. The PI points to the reduction in droplet size homogeneity by reaching values up to 1.3 on day 12. In addition to the freeze–thaw cycles, after the preparation (T0) and after T12, the formulations were also centrifuged at 12,000 rpm (for 30 min). Therefore, after centrifuging at T0, the phase separation of formulations F6, F13, F14, and F15 (all composed of SA) was observed. This behavior was influenced by the surface properties of the droplets, which showed low interaction ability (inactive particle fillers, Figure 2c, I) with the polymer chains constituting the gel [54]. The reduced chemical affinity with the polymeric matrix may be associated with a smaller amount of natural compounds capable of acting as surfactants, which can play a great role in the strengthening of the droplet surface and contribute to its stability. Furthermore, SA presents low viscosity when compared to other explored vegetable oils (Figure 3), which may facilitate the mobility and coalescence of the droplets into these systems [55].

Phase separation was observed after T12 followed by centrifugation for formulations F4, F5, F22, F23, and F24 (SA in the presence of PE). The addition of PE provided suitable surface tension, which improved the stability of the systems until day 12. Hence, droplets composed of the PE+SA indicated that better chemical interactions were established with the polymeric matrix (Figure 2c, II and Figure S1). However, the high trend of coalescence for SA preparations discouraged further studies. On the other hand, the combination of PE and PF or AN oil phase led to highly stable droplet size (low PI values) under thermal and temporal dynamics, reinforcing a potential stabilizing effect of PE (with or without CUR) components, as proposed in Figure 2c, II. The centrifugal inertial shape and the consequent shear stress quote droplets deformations, with small collision and coalescence for systems containing AN and PF (active droplets, with interface/oil component chemical interaction, Figure 2c, II and III). Thus, emulgels composed of AN oil presented reduced variation in globule size, with F9 and F17 displaying the smallest size of globules. Although most AN systems showed an increment of the droplet size, their variation range was smaller than that observed for PF systems. AN oil presents low chemical unsaturations in its structure and, consequently, high viscosity is be observed in Figure 3, hindering the oil transition through the polymeric mesh, also preventing coalescence. Therefore, AN oil showed to be the most stable vegetable oil in the emulgels studied.

Although the droplet size increased in most of the systems, the physical integrity of all systems displayed in Figure 2 was maintained, remaining as a single phase. The variations caused by repetitive thermal dynamics were subtle and would not be detrimental for therapeutic purposes.

### 2.3. Texture Profile Analysis (TPA)

**Figure 4:**
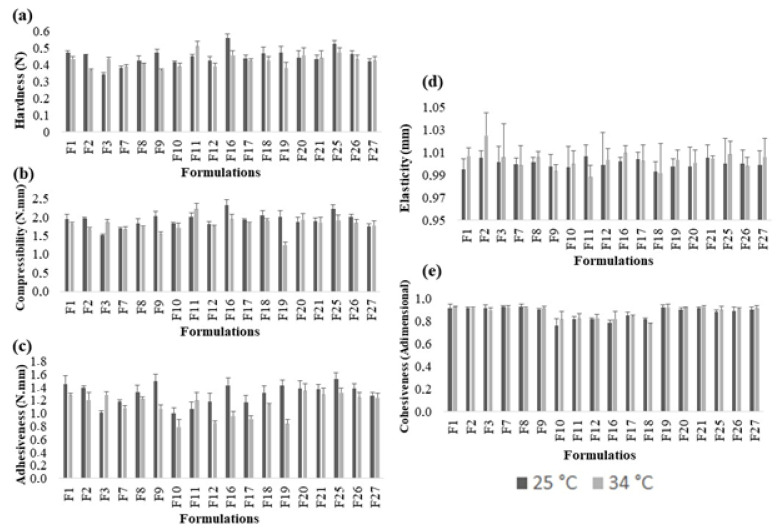
The effect of temperature, polymer type, vegetable oil type, and bioactive agent composition on TPA properties of the emulgels at 25 °C and 34 °C: (**a**) hardness; (**b**) compressibility; (**c**) adhesiveness; (**d**) elasticity; (**e**) cohesiveness. Each bar graph represents the average, and the relative standard deviation was less than 10% (n = 3).

The TPA makes the evaluation of the organization and the interactions among the components of formulation possible and can guarantee information about the physical structure of the emulsion system. It also allows the system’s responses under the external mechanical stimulus to be understood, showing their ability to undergo reversible and irreversible deformations [56,57,58,59]. In this study, hardness, compressibility, adhesiveness, elasticity, and cohesiveness properties were investigated for each formulation (Figure 4).

Hardness is the force required to deform a sample and may represent the work/stress necessary to remove the emulsion system from a container and to apply it to the target site, for example [59]. The effect of PC was statistically significant in the hardness of the formulations (*p* < 0.05), with a hardness decrease. We have demonstrated hydrogels containing pure C974P, and PC demonstrated higher hardness values at 25 °C than at 37 °C [21], with the opposite observed when composing the emulgels. This may be linked to the ability of the droplets to interact with the polymer interface, which leads to point destructuring effects [54]. For the independent variable type of oil, a significant effect for both levels (*p* < 0.05) was observed. Moreover, the temperature of 34 °C also promoted a decrease in hardness due to the slight thermodependent properties of the acrylic acid derivatives. These polymers exhibit weakness of electrostatic repulsions as the temperature increases, which sustain the extended polymeric network. Similarly, compressibility is the force per unit of time required to deform the sample at the first compression, determining the removal of the product from the packaging material and its spreadability at the site of action [58]. The presence of C974P provided an increase in the compressibility of the systems when compared to PC.

For adhesiveness, the different polymers used displayed no significant difference (*p* > 0.05). Considering the independent variable oil type, both showed a significant effect (*p* < 0.05). Among the bioactive agents, formulations containing PE and PE+CUR showed a significant increase in adhesiveness. The increase in temperature significantly decreased the adhesiveness of the formulations (*p* < 0.05). Elasticity, in turn, is the ability of the formulation to stretch and recover its original structure after a deformation being applied and removed [58,59]. The effects of all the independent factors (polymer type, oil type, bioactive agent, and temperature) were not significant in the elasticity of the preparations (*p* < 0.05). In comparison to pure C934P [60], C974P [61], and PC [4] dispersions, the addition of both vegetable oils and active agents significantly improved the hardness and adhesiveness of the preparations (*p* < 0.05).

Cohesiveness is a mechanical textural parameter that can also influence the performance of the system. It is mainly related to the restructuring ability of the emulsion systems after successive shear stresses are applied. High cohesiveness values mean high organization and performance of the product at the site of application. Thus, under the conditions studied, the formulations presented full ability to recover their initial structure after stress being removed [3,58,62]. Overall, the emulgels systems displayed good cohesiveness, with values ranging from 0.8154 to 0.9266 at 25 °C and from 0.7803 to 0.9265, at 34 °C. However, for the independent variables (polymers, oils, and temperatures), there was no significant difference in this parameter (*p* > 0.05). Therefore, only the bioactive type demonstrated a significant influence on cohesiveness, with CUR presenting a significant influence by decreasing this mechanical characteristic (*p* < 0.05).

The surface response graphs for each parameter of TPA displayed different trends relying on the temperature and vegetable oil used (Figures S2–S5). The surfaces were adjusted to the experimental data by multiple adjusted determination coefficients (R^2^_adj_), which demonstrated higher values for hardness and compressibility overall. The response surface was in agreement with the previous stability data and showed a higher droplet/interface interaction trend for the systems composed of PE and PE+CUR combined with PF and AN. For PF emulgels, at 25 °C, the highest hardness (R^2^_adj_ = 0.8075), compressibility (R^2^_adj_ = 0.6193) and adhesiveness (R^2^_adj_ = 0.5229) values were observed for any bioactive that used C934P or C974P. However, the most elastic preparation was composed of PC, PE+CUR. At 34 °C (Figure S3), the opposite was observed, with the highest values of hardness, compressibility (R^2^_adj_ = 0.3746) and adhesiveness (R^2^_adj_ = 0.08379) being observed for preparations containing any poly(acrylic acid) derivative and PE as bioactive. Cohesiveness, on the other hand, was improved for C934P and PE+CUR.

Considering emulgels containing AN, at 25 °C (Figure S4), a similar profile was noticed for hardness (R^2^_adj_ = 0.9044), compressibility (R^2^_adj_ = 0.8654) and adhesiveness (R^2^_adj_ = 0.5007). Furthermore, high values of these parameters were observed for formulations containing C934P and PE+CUR in addition to PC and PE. Cohesiveness (R^2^_adj_ = 0.0358), on the other hand, exhibited the highest values for C934P polymer and PE bioactive. However, at 34 °C (Figure S5), the highest values for hardness (R^2^_adj_ = 0.7883), compressibility (R^2^_adj_ = 0.5714) and adhesiveness (R^2^_adj_ = 0.2413) were attained by emulgels comprising C934P and PE+CUR as bioactive agents.

### 2.4. Softness

**Figure 5:**
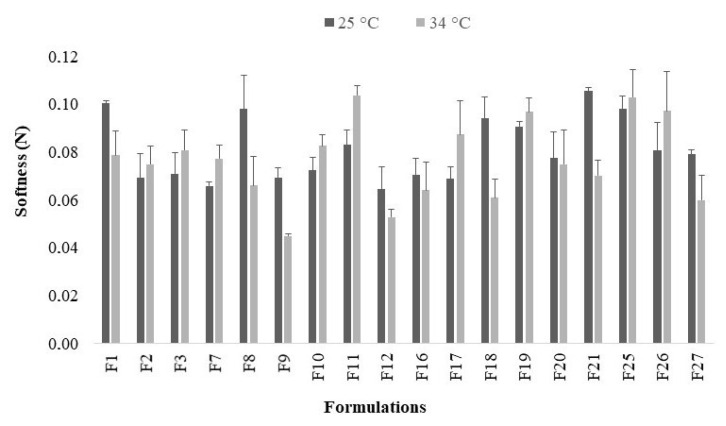
Determination of softness index (N) of the emulsion systems at 25 °C and 34 °C (n = 3).

The softness analysis is complementary to TPA, being different by using a conical perspex probe with a 45° angle. This ensures the sample presents a plastic behavior by reducing the viscosity during the penetration of the probe, with a greater contact area [56]. The maximum force required for penetration into the sample is determined and correlated with the smoothness degree. The analyses were performed at 25 °C and 34 °C (Figure 5) to obtain the softness index at room and body temperature, in order to simulate this behavior at the intended application site [4]. All systems showed a good softness profile, namely as a low force (≤0.10 N), both being considered soft samples [4,56]. Compared to the poly(acrylic acid) derivatives’ raw dispersions, the softness values were not different when the organic and bioactive compounds were added to the systems [3,61].

Formulations containing AN oil (at 25 °C) did not demonstrate a significant difference in softness values using the three polymers or the three combinations of bioactive agents (*p* < 0.05). However, at 34 °C, as also observed for emulgels containing the PF oil, PC demonstrated significant differences against C934P (*p* = 0.000477) and C974P (*p* = 0.000936). 

The surface response plots (Figure S6) displayed that the most extensive softness index (high rigidity) was obtained for systems composed of C934P as polymers, quoting its highly cross-linked structure in comparison to the other poly(acrylic acid) derivatives. For instance, systems containing AN (at 25 °C and 34 °C) reached high rigidity using C934P and PE+CUR as bioactive agents. For emulgels composed of PF, at 25 °C, high index values were observed for preparations with C934P and with only PE as a bioactive compound, in addition to PC with PE+CUR. Meanwhile, at 34 °C, the index followed the same trend of emulgels containing AN, reaching high values for preparations containing C934P and PE+CUR. The surfaces were adjusted to the experimental data by R^2^_adj_, varying between 0.6968 and 0.7443. Through regression analysis, it is observed that this model may explain around 70% of the experimental data.

### 2.5. Evaluation of Bioadhesive Properties

**Figure 6:**
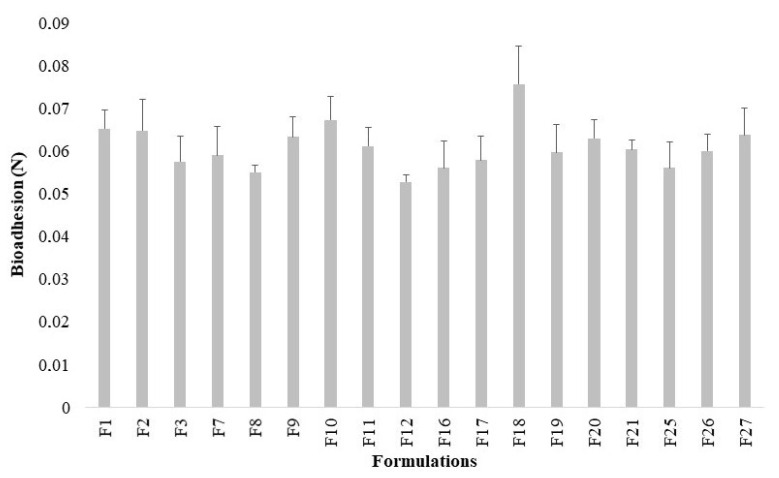
Effect of polymeric content, vegetable oils and different bioactive agents in bioadhesion of the emulsion systems at 34 °C. The plots represent the mean plus standard deviation, and the relative standard deviation was less than 12% (n = 3).

All formulations displayed similar force values necessary to detach the pig skin from the sample surface (Figure 6). The effects of polymer type, vegetable oils, and different bioactive agents were not statistically significant in bioadhesion (*p* > 0.05).

Compared to some thermoresponsive hydrogels containing C934P [60], C974P [61], and PC [4], the bioadhesive force of the studied emulgels was lower than the mucoadhesive force observed for most polymer blends composed of poloxamer 407 and the same poly(acrylic acid) derivatives, suggesting higher interactions of these polymers with mucin than to the skin. However, combined with vegetable oils, the polymers may constitute systems with higher permeability than gels. Raw solutions of 2% (*w*/*w*) C974P or PC [21] demonstrated similar bioadhesion values by tensile strength method, using porcine ear skin (values around 6 g = 0.06 N), for both polymer gels. Therefore, acting as a matrix for vegetable oils and the studied bioactive agents into the emulgels, poly(acrylic acid) derivatives may perform similar bioadhesive behavior to their respective hydrogels.

### 2.6. Rheological Analysis

#### 2.6.1. Continuous Shear

A range of parameters can be evaluated by continuous (flow) shear rheometry, such as ease of administration and interactions between the formulation constituents and their structuring [57,59,63,64]. The emulsion systems showed non-Newtonian and pseudoplastic flow behavior at both temperatures (Table 1), with flow behavior index (*n*) similar to the reported in the literature for hydrogels containing the same poly(acrylic acid) derivative [21,65]. Their consistency index (*K*) was dependent on the presence of the polymer PC, bioactive CUR, and the different types of vegetable oils (*p* < 0.05). Emulgels containing C974P and C934P demonstrated increased *K* values since they are derivatives with a high cross-linking degree [21,65], which may foster improved structure to the oily phase. It was observed that the presence of CUR significantly increased the *K* values (*p* = 0.000111), while emulgels with PE showed lower *K* at both 25 °C and 34 °C. This behavior may be associated with the composition of the droplets considering the preferential solubilization of CUR and PE in this phase. In previous studies, the hydrophobic CUR has shown its ability to be in monomeric form within the oil droplets (data not shown). On the other hand, PE comprises several complex components that may be distributed between the aqueous and oily phases of the emulgel, leading to reduced *K* values [33,42,66]. Vegetable oils also displayed a significant effect for this parameter with (*p* = 0.000174), agreeing with their respective viscosity as shown in Figure 3, AN preparations presented higher *K* values overall.

Considering the flow behavior index of the emulgels, significance was observed between C934P and PC polymers, the presence of CUR, and the oils (*p* < 0.05). Emulgels composed of PC displayed *n* values higher than C934P, suggesting that a system should be built with lower structuring. However, the yield value (*τ*_0_) and the hysteresis area were almost identical at 25 °C and 34 °C for most of the systems. All formulations showed yield values ranging from 12 Pa to 285 Pa; thereby, most of them showed resistance to flow when low stress was applied. Highly viscous materials present better resistance to the shear found in the skin and the systems start to flow due to a weakening of the fluid-structure [10,59,67]. High *τ*_0_ can promote increased residence time of the drug at the site of action, also avoiding the destruction of the emulgel structure [10]. Formulations composed of C934P, PF or AN oil, and both bioactive agents, exhibited significant differences (*p* < 0.05) for *τ*_0_ at 25 °C and 34 °C.

For the hysteresis area, the type of bioactive agent was a statistically significant factor (*p* < 0.05). The hysteresis area is the response of the formulation subjected to shear stress. Increasing the shear rate over time can result in a maximum shear value. Nevertheless, when the shear rate is reduced, the process is reversed and a decrease in shear rate fosters a region bounded by the up and down curves, called thixotropy [10,67]. Overall, the thixotropy indicated a good restructuring ability (low hysteresis area) for most emulsion systems (Figures S7 and S8), also benefitting the spreadability of the emulgel throughout the skin together with the pseudoplastic behavior [1]. However, formulations F10, F12, F17 and F18, which have CUR in common, showed some disorganization degree (higher hysteresis area). The presence of PE resulted in a better structuring for the systems, which may be useful for topical applications and complies with the proposed stability mechanism, considering PE helps the stabilization of the active droplet oils (Figure 2, II and III).

The formulations become more fluid when a force is applied (simulating pumping, agitation, topical and local administration, for example). However, they can recover their initial viscosity over a resting period [10,42,67]. Formulations containing only CUR showed the highest thixotropy area indicating that the presence of this bioactive resulted in a longer period of time for the restoration of the initial molecular configuration. These formulations may be more sensitive to breakage by high shear rates, which may be less advantageous in aiding the retention of the emulgel at the desired site.

#### 2.6.2. Viscoelastic Measurements

**Figure 7:**
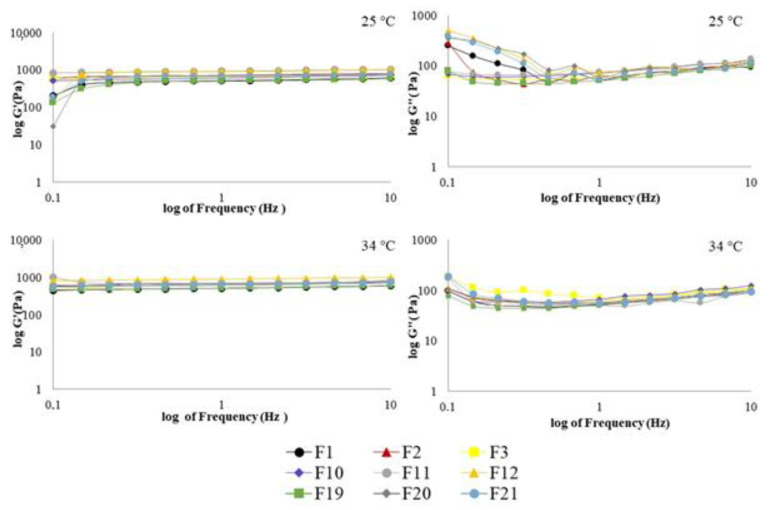
Elastic modulus (G’) and viscous modulus (G’’) as a function of frequency of formulations containing PF oil. Each point is the mean of at least three replicates. Standard deviations have been omitted for clarity; however, in all cases, the relative standard deviation of replicate analysis was less than 10%.

**Figure 8:**
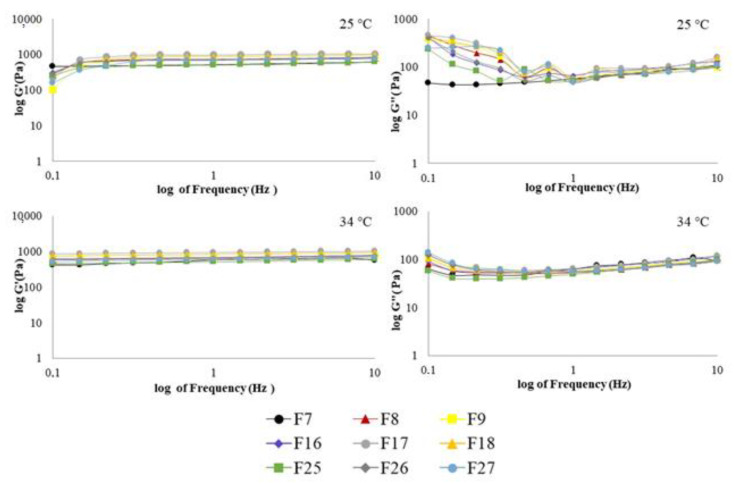
Elastic modulus (G’) and viscous modulus (G’’) as a function of frequency of formulations containing AN oil. Each point is the mean of at least three replicates. Standard deviations have been omitted for clarity; however, in all cases, the relative standard deviation of replicate analysis was less than 10%.

All formulations exhibited elastic modulus (G’) greater than the viscous modulus (G”) throughout the most frequencies analyzed, at all temperatures, with viscoelastic systems being highly structured [68,69]. Viscoelasticity is a desirable characteristic, that has been demonstrated to improve the retention of the preparations at the application site [3,59,65,70]. Although G’ was not dependent on the frequency sweep, G” values changed over the frequency range evaluated, displaying relative standard deviation values up to 10% (Tables S2–S7). Both PF and AN systems (PF, Figure 7 and AN, Figure 8) suggest loops and high crosslink density among their constituents. The polymer type (CP34P, C974P, PC), oil type (PF, AN), bioactive type (PE, CUR, PE+CUR), and the different temperatures (25 °C and 37 °C) utilized did not significantly affect the viscoelastic properties (*p* > 0.05).The dynamic viscosity (η’) of these formulations decreased as the frequency increased (Figure S9), with AN systems demonstrating higher η’ values than PF preparations, agreeing with the viscosity of each oil.

The loss tangent (tan δ) slightly changed with increasing frequency (Figures S10 and S11). A comparison among formulations containing AN, at 25 °C (Figure S10 and Figure 8), demonstrated that F7 (composed of C934P, AN oil and PE) was unique in displaying tan δ values less than one over the entire frequency sweep analyzed. Particularly, when it is compared to F16 and F25, which present the same polymer and oil composition, improved viscoelastic characteristics are revealed for F7. At low frequencies and 25 °C, F16 and F25 demonstrated elastoviscous behavior, reflecting a reduced interaction among the components of these preparations. Increasing the oscillatory frequencies, there was improved interaction of the constituents, since a viscoelastic behavior is noted. Previous studies have shown the enhancement of viscoelastic characteristics of hydrogel systems composed of C934P and PE [24,42]. Emulgels comprising PE have already been reported about their higher ability to form more structured emulsion systems, due to the greater content of resin and gum of this bioactive agent [33]. Moreover, the chemical and physicochemical characteristics of the AN oil (e.g., high viscosity) contributed to improve the G’ modulus in relation to loss modulus, resulting in an emulsion system with predominant viscoelastic behavior overall range of frequency analyzed.

### 2.7. Physicochemical Properties Correlation

Linear regression is used to analyze the correlation between two variables [71,72]. Therefore, it was used to evaluate the linear correlation and droplet size and mechanical and rheological properties with determination coefficient (R^2^) values ranging from 0.0017 to 0.9617 for the systems at 25 °C (Table 2).

Negative correlations were not observed. Rheological, mechanical and droplet size parameters were not strongly correlated for emulgels systems, displaying R^2^ frequently lower than 0.1. However, the parameters hardness and compressibility showed good correlation (R^2^ = 0.9617), complying with the data reported elsewhere [73].

While good correlations between rheological and mechanical parameters have not been demonstrated for most of the properties studied, it is important to consider that rheological responses may be more perceptible and able to detect interactions among the components of the semi-solid systems more effectively, and more able to investigate the organization of formulations on a nanometric scale [72,74].

## 3. Conclusions

The emulgels composed of passion fruit or andiroba oil showed physicochemical stability, and andiroba oil preparations were the most stable. The polymers Carbopol 934P and polycarbophil resulted in suitable physical structures for dispersion of the vegetable oils and bioactive agents. Gels composed of one of these poly(acrylic acid) derivatives demonstrated the best smoothness and mechanical properties for topical application. The formulations exhibited pseudoplastic flow behavior and viscoelasticity relying on the bioactive added. The formulations were soft, indicating ease of application. Therefore, the utilization of one of these polymers and passion fruit or andiroba oil constitutes a good strategy for developing highly stable emulgels for local delivery of the bioactive agents investigated, joining hydrogel and emulsion properties. Systems containing andiroba oil and Carbopol 934P or polycarbophil showed promising formulations for further investigations as topical drug delivery systems for curcumin and/or propolis administration on the skin and mucous membranes. 

## 4. Materials and Methods

### 4.1. Materials

Carbopol 934P^®^ (C934P) was purchased from BF Goodrich (Brecksville, OH, USA). Carbopol 974P^®^ (C974P) and polycarbophil (PC) were kindly received from Lubrizol (Sao Paulo, Brazil). Sweet almond oil (SA) was purchased from All Chemistry (Sao Paulo, Brazil), Passion fruit oil (PF) was purchased from Amazon Oils (Ananindeua, Brazil) and Andiroba oil (AN) was purchased in a popular market in the Brazilian Amazonian region (Ver o Peso market, Belem, Brazil). Brazilian green propolis (PRP) was obtained from an apiary of *Apis mellifera* L. bees, located inside a eucalyptus reserve, surrounded by native forest with a predominance of *Baccharis dracunculifolia* (Asteraceae), in the northwest of Parana state. Curcumin C3 complex^®^ was received from Sabinsa^®^ (West Windsor, NJ, USA) and triethanolamine, used as a neutralizing agent, was purchased from Galena (Campinas, Brazil). Purified water was obtained in-house using a water purification system (Evoqua Water Technologies, Pittsburgh, PA, USA). This research was registered, in Brazil, by SISGEN N° A098049 (AN) and N° AC7A2F5 (PRP).

### 4.2. Preparation of Emulgel Systems

Propolis extract propolis (PE) (30% *w*/*w*) was prepared using ethanol 96% (*v*/*v*), by turbo-extraction, at 3500 rpm for 15 min, with two rest periods of 5 min. After extraction, the extractive solution PE was obtained by filtration (paper filter grade 3) [63,67].

The emulsion systems were prepared following a full factorial design 3^3^, with three independent factors (type of polymer, type of vegetable oil, and bioactive agent), and at three levels (−1, 0 and +1) (Table 3). First, the polymer (1%, *w*/*w*) was dispersed in purified water under mechanical agitation at 200 rpm. After the complete dispersion of the polymer, CUR (0.1%, *w*/*w*) was added and then the pH was neutralized with triethanolamine [24,42,64]. Then, PE (8%, *w*/*w*) was added and, finally, the oil (8%, *w*/*w*) (SA, PF or AN) under constant mechanical agitation. All formulations were hermetically stored, in vials, for at least 24 h before analysis.

### 4.3. Physicochemical Stability Study

The formulations were evaluated for preliminary physicochemical stability by freezing (−5 ± 2 °C) and thawing (40 ± 2 °C) cycles, over 24 h each, for 12 days (from time T0 to time T12), with a total of six cycles [75,76,77]. Afterwards, the systems were evaluated as color and appearance changes. In addition, all formulations were evaluated as phase separation when submitted to centrifuging (12,000 rpm during 30 min) just after T0 and T12. At the end of each cycle, droplet size analysis was carried out, using an optical microscope (Kozo Optics, Nanjing, China) and the Image Pro-Plus 4.5.0.29 software (Media Cybernetics Inc., Rockville, MD, USA). The polydispersity index (PI) was calculated using the following equation [63,64,74,75]:

(1) $$PI=\frac{\left(D_{90\%}-D_{10\%}\right)}{D_{50\%}}$$

where *D*_90%_, *D*_50%_, and *D*_10%_ correspond to the cumulative mean diameter of 90%, 50%, and 10% of the droplets, respectively.

### 4.4. Viscosity Study

The viscosity of the SA, AN and PF oils was measured by viscosimeter (Visco Star Plus, Fungilab, Spain) fitted with disc spindles at 25 °C, 34 °C and 40 °C. All measurements were carried out using four replicates [78,79].

### 4.5. Texture Profile Analyses (TPA)

The texture profile of the formulations was determined using a TA-XTplus texture analyzer (Stable Micro Systems, Surrey, UK). For the TPA analysis, the equipment was placed in compression mode, and the samples were subjected to double compression by an analytical probe, with 10 mm of diameter, at a set rate of 2 mm/s, and at a depth of 15 mm. A delay period of 15 s between the first and the start of the second compression was allowed between the compression cycles. The analyses were carried out at 25 °C and 34 °C. The parameters hardness (maximum force during compression), compressibility (the work necessary to deform during the first pass of the probe), adhesiveness (work necessary to overcome tensile forces between probe surface and sample), elasticity (ability to stretch and return to its original size and shape) and cohesiveness (work necessary to join the surface of the sample and the surface of the probe) were derived from a graph of force-time and force-distance parameters. For each analysis, at least three replicates were performed [3,5,42,80].

### 4.6. Determination of Softness

The softness index was determined by using the same texture analyzer equipment previously described, at 25 °C and 34 °C, in compression mode and equipped with a conical Perspex probe (P/45C). The sample (22.5 g) was transferred to a glass beaker (50 mL), preventing air bubbles. Afterward, the probe was compressed into the sample (10 mm) at a speed of 1mm/s for 25 s. The softness index was determined as the maximum force required for the probe to penetrate into the sample, and it was calculated as the maximum value in a graph between force and distance [3]. The analysis was performed at least in three replicates for each formulation.

### 4.7. Ex Vivo Evaluation of Bioadhesive Strength

The same texture analyzer above described was used in tension mode. The bioadhesive strength was evaluated by measuring the force required to separate the emulgels from the surface of porcine pig ear skin [42,81]. Firstly, ear skin samples were taken from young, white, freshly slaughtered pigs (from a slaughterhouse authorized by the Ministry of Agriculture for consumption). The pig ears were cleaned and the posterior skin of the ear was removed using a surgical scalpel. Ears presenting warts, wounds, or hematomas were not used [81].

Before the analysis, a polypropylene vial was used to support the skin, exposing a circular skin surface of 8 mm diameter from the vial by 2 mm. This set was attached to a mobile cylindrical probe (P/6) using double-sided adhesive tape. The emulsion systems were previously packed in glass vessels and stored at 34 °C. The analytical probe lowered until the skin reach the surface of the emulgel. A downward force of 0.1 N was applied for a specific period time (30 s) to ensure close contact between the skin and the samples. Then, the probe was moved upwards (1.0 mm/s), and the required force to detach the skin from the formulations was determined as the maximum value in the relationship between force and distance. All measurements were performed at least three times [57,60].

### 4.8. Rheometry

Rheometry of the formulations was performed using a MARS II rheometer (Thermo Haake Fisher Scientific Inc., Newington, Germany) with controlled gradient and stress at 25 and 34 ± 0.1 °C. A parallel steel cone-plate (35 mm diameter; gap 0.105 mm) was utilized. The samples were carefully placed on the bottom plate, ensuring minimal formulation shear and allowing a rest time of 1 min before each determination.

#### 4.8.1. Continuous Shear (Flow)

Continuous shear analysis was performed in flow mode over a range of shear rates from 0 to 2000 s^−1^. The shear rate was increased over a period of 150 s, kept at the upper limit during 10 s, and then decreased over 150 s. At least three replicates of each sample were analyzed. The hysteresis area of each system was also calculated using RheoWin 4.10.0000 (Haake^®^) software. The upward flow curves were modeled using the Ostwald-de-Waele equation [42,64]:

(2) $$\tau =K.{\dot{\gamma }}^{n}$$

where *τ* is the shear stress (Pa), *K* is the consistency index [(Pa.s)^n^], $\dot{\gamma }$ is the shear rate (s^−1^), and *n* is the flow behavior index (dimensionless).

The yield value of each formulation was determined by Casson and Herschel–Bulkley rheological models according to Equations (3) and (4), respectively [82]:

(3) $$\tau =\sqrt[{n}]{\left(\tau_{0}^{n}+(\dot{\gamma }.n_{p}\right){)}^{n}}$$

where *τ* is the shear stress (Pa), *n* is the flow behavior index (dimensionless), *τ*_0_ is yield stress (Pa), $\dot{\gamma }$ is the rate of shear (s^−1^) and *η_p_* is Casson plastic viscosity.

(4) $$\tau =\tau_{0}+K.{\dot{\gamma }}^{n}$$

where *τ* is the shear stress (Pa), *τ*_0_ is yield stress (Pa), *K* is the consistency index [(Pas)^n^], $\dot{\gamma }$ is the rate of shear (s^−1^) and *n* is the flow behavior index (dimensionless).

#### 4.8.2. Oscillatory Rheometry

After fitting linear viscoelastic region (LVR), the frequency sweep was performed from 0.1 Hz to 10.0 Hz [3,80,81,83]. The elastic modulus (G’), viscous modulus (G”), dynamic viscosity (η’), and loss tangent (tan δ) were measured by using the RheoWin 4.10.0000 (Haake^®^) software. At least three replicates for each sample were analyzed.

### 4.9. Statistical Analysis

The effects of poly(acrylic acid) derivatives types (C934P, C974P, PC) and type of vegetable oil and temperature on textural, softness and rheological properties of formulations containing PE, CUR, PE+CUR were statistically evaluated using a design of experiment (DoE) and compared by analysis of variance (ANOVA).

The experiments were randomized, in order to minimize the influence of unexplained variability in the responses. In all cases, individual differences between means were identified using Turkey’s honestly significant difference test. The linear correlation between droplet size, hardness, compressibility, tan δ, hysteresis area and consistency index at 25 °C were determined by linear regression analysis. A value of *p* < 0.05 was considered statistically significant and the Statistica software version 10.0 (StatSoft Company, Tulsa, OK, USA) was used throughout.

## Figures and Tables

**Table 1:** The effects of different types of polymer (C934P, C974P, PC), oil (PF, AN) and bioactive agent combinations (PE, CUR, PE+CUR) on the consistency index (*K*) and rheological exponent (*n*) of emulgels at 25 and 34 °C.

Formulations	*K* (Pa.s)		*n* (Dimensionless)	
	25 °C	34 °C	25 °C	34 °C
F1	68.45 ± 0.35	67.65 ± 0.29	0.35 ± 0.00	0.34 ± 0.00
F2	83.06 ± 6.39	82.06 ± 6.74	0.33 ± 0.00	0.33 ± 0.00
F3	60.78 ± 0.96	60.78 ± 0.96	0.34 ± 0.00	0.34 ± 0.00
F7	71.39 ± 0.19	71.39 ± 0.19	0.33 ± 0.00	0.33 ± 0.00
F8	75.17 ± 4.22	70.22 ± 1.06	0.33 ± 0.00	0.33 ± 0.00
F9	86.72 ± 7.06	69.71 ± 0.99	0.28 ± 0.02	0.33 ± 0.00
F10	191.65 ± 1.25	191.65 ± 1.25	0.16 ± 0.04	0.16 ± 0.04
F11	67.84 ± 1.06	67.84 ± 1.06	0.35 ± 0.00	0.35 ± 0.00
F12	71.55 ± 4.89	61.27 ± 2.50	0.36 ± 0.01	0.35 ± 0.04
F16	107.07 ± 2.53	110.67 ± 4.51	0.29 ± 0.00	0.28 ± 0.00
F17	138.77 ± 6.29	128.00 ± 7.10	0.25 ± 0.00	0.25 ± 0.00
F18	103.00 ± 2.08	77.72 ± 6.05	0.28 ± 0.00	0.31 ± 0.01
F19	71.37 ± 1.44	74.53 ± 0.17	0.34 ± 0.00	0.32 ± 0.00
F20	85.64 ± 6.31	80.71 ± 1.89	0.32 ± 0.01	0.32 ± 0.00
F21	66.76 ± 2.46	62.64 ± 2.73	0.34 ± 0.00	0.35 ± 0.00
F25	66.27 ± 1.67	65.78 ± 0.54	0.34 ± 0.01	0.33 ± 0.00
F26	75.47 ± 2.22	75.85 ± 6.78	0.33 ± 0.00	0.32 ± 0.01
F27	73.35 ± 2.14	54.65 ± 0.77	0.33 ± 0.00	0.35 ± 0.00

**Table 2:** Correlation between rheological (consistency index, hysteresis area and tan δ), mechanical properties (hardness and compressibility) and droplet size of emulgels systems composed of polymers (C934P, C974P, PC), different vegetable oils (PF, AN) and bioactive agent combinations (PE, CUR, PE+CUR) at 25 °C.

Correlation Parameter	R^2^-Value
Droplet size/Hardness	0.0570
Droplet size/Compressibility	0.0932
Droplet size/Tan δ	0.2954
Droplet size/Hysteresis area	0.0170
Hardness/Compressibility	0.9617
*K* */Hardness	0.0017
*K* */Compressibility	0.0102

* Consistency index.

**Table 3:** Matrix of factorial design 3^3^ for the emulgel systems containing three types of polymers (C934P, C974P, PC), vegetable oils (PF, SA, AN) and also three combinations of bioactive agents (PE, CUR, PE+CUR).

Independent Variables	Levels		
	Low (−1)	Central (0)	High (+1)
X_1_ = Polymer type	C934P	C974P	PC
X_2_ = Oil type	PF	SA	AN
X_3_ = Bioctive agent	PE	CUR	PE+CUR
Standard run (formulations)	X_1_	X_2_	X_3_
F1	−1	−1	−1
F2	0	−1	−1
F3	+1	−1	−1
F4	−1	0	−1
F5	0	0	−1
F6	+1	0	−1
F7	−1	+1	−1
F8	0	+1	−1
F9	+1	+1	−1
F10	−1	−1	0
F11	0	−1	0
F12	+1	−1	0
F13	−1	0	0
F14	0	0	0
F15	+1	0	0
F16	−1	+1	0
F17	0	+1	0
F18	+1	+1	0
F19	−1	−1	+1
F20	0	−1	+1
F21	+1	−1	+1
F22	−1	0	+1
F23	0	0	+1
F24	+1	0	+1
F25	−1	+1	+1
F26	0	+1	+1
F27	+1	+1	+1

Carbopol 934P^®^ = C934P; Carbopol 974P^®^ = C974P; Polycarbophil = PC; Passion fruit oil = PF; Sweet almond oil = SA; Andiroba oil = AN; Propolis extract = PE; Curcumin = CUR.

## Supplementary Materials

The following are available online at https://www.mdpi.com/article/10.3390/gels7030120/s1, Figure S1: Phase separation of the systems during the physicochemical stability analysis at time zero (a): F6, F13, F14, F15; and after 12 days (b): F4, F5, F22, F23, and F24, Figure S2: Response surface plots of mechanical properties (hardness, compressibility, adhesiveness, elasticity and cohesiveness) of PF emulgels influenced by different polymers (C934P, C974P, PC), different type of bioactive (PE, CUR, PE+CUR) at 25 °C. The color scale is indicated in each figure and shows the isoparametric values, Figure S3: Response surface plots of mechanical properties (hardness, compressibility, adhesiveness, elasticity and cohesiveness) of PF emulgels influenced by different polymers (C934P, C974P, PC), different type of bioactive (PE, CUR, PE+CUR) at 34 °C. The color scale is indicated in each figure and shows the isoparametric values, Figure S4: Response surface plots of mechanical properties (hardness, compressibility, adhesiveness, elasticity and cohesiveness) of AN emulgels influenced by different polymers (C934P, C974P, PC), different type of bioactive (PE, CUR, PE+CUR) at 25 °C. The color scale is indicated in each figure and shows the isoparametric values, Figure S5: Response surface plots of mechanical properties (hardness, compressibility, adhesiveness, elasticity and cohesiveness) of AN emulgels influenced by different polymers (C934P, C974P, PC), different type of bioactive (PE, CUR, PE+CUR) at 34 °C. The color scale is indicated in each figure and shows the isoparametric values, Figure S6: Response surface plots of softness as a function of different polymers (C934P, C974P, PC), different type of bioactive (PE, CUR, PE+CUR) and vegetable oils (PF or AN) for emulgels systems, at 25 and 34 °C. The color scale is indicated in each figure and shows the isoparametric values, Figure S7: Flow curves of the formulations containing PF at different temperatures. Closed symbol represents the upcurve and open symbol represents the downcurve. Standard deviations have been omitted for clarity; however, in all cases, the relative standard deviation of replicate analysis was less than 10%, Figure S8: Flow curves of the formulations containing AN at different temperatures. Closed symbol represents the upcurve and open symbol represents the downcurve. Standard deviations have been omitted for clarity; however, in all cases, the relative standard deviation of replicate analysis was less than 10%, Figure S9: Viscosity ἠ as a function of frequency of formulations without PF and AN oil at temperatures 25 and 34 °C. Each point is the mean of at least three replicates. Standard deviations have been omitted for clarity; however, in all cases, the relative standard deviation of replicate analysis was less than 10%, Figure S10: Tan δ as a function of frequency of formulations containing PF at temperatures of 25 (A) and 34 °C (B). Each point is the mean of at least three replicates. Standard deviations have been omitted for clarity; however, in all cases, the relative standard deviation of replicate analysis was less than 10%, Figure S11: Tan δ as a function of frequency of formulations containing AN at temperatures of 25 (A) and 34 °C (B). Each point is the mean of at least three replicates. Standard deviations have been omitted for clarity; however, in all cases, the relative standard deviation of replicate analysis was less than 10%, Table S1: Mean globule size and polydispersity index (PI) of dispersed phase of the emulgels containing PF or AN oils just after the preparation (T0) and after 12 days (T12) of the ice-thawing cycle, Table S2. Relative standard deviation (%) of emulgels systems F1, F2 and F3, containing PF and PE: elastic modulus (G’) and viscous modulus (G”) at 25 and 34 °C, Table S3. Relative standard deviation (%) of emulgels systems F7, F8 and F9, containing AN and PE: elastic modulus (G’) and viscous modulus (G”) at 25 and 34 °C, Table S4: Relative standard deviation (%) of emulgels systems F10, F11 and F12, containing PF and CUR: elastic modulus (G’) and viscous modulus (G”) at 25 and 34 °C, Table S5: Relative standard deviation (%) of emulgels systems F16, F17 and F18, containing AN and CUR: elastic modulus (G’) and viscous modulus (G”) at 25 and 34 °C, Table S6: Relative standard deviation (%) of emulgels systems F19, F20 and F21, containing PF and PE+CUR: elastic modulus (G’) and viscous modulus (G”) at 25 and 34 °C, Table S7: Relative standard deviation (%) of emulgels systems F25, F26 and F27, containing PF and PE+CUR: elastic modulus (G’) and viscous modulus (G”) at 25 and 34 °C.
