# Supplementary material for: Emulgels Containing Propolis and Curcumin: The Effect of Type of Vegetable Oil, Poly(Acrylic Acid) and Bioactive Agent on Physicochemical Stability, Mechanical and Rheological Properties
Source: Gels. 2021 Aug 12;7(3):120. doi: 10.3390/gels7030120 (PMC8396026; doi:10.3390/gels7030120)
Supplement: Supplementary file 1 [file gels-07-00120-s001.zip › gels-1265031-supplementary.pdf]

Supplementary Material

Emulgels Containing Propolis and Curcumin: The Effect of Type of Vegetable Oil, Poly(Acrylic Acid) and Bioactive Agent on Physicochemical Stability, Mechanical and Rheological Properties

Rafaela Said dos Santos [1], Jéssica Bassi da Silva [1], Hélen Cássia Rosseto [1], Camila Felix Vecchi [1], Katieli da Silva Souza Campanholi [2], Wilker Caetano [2] and Marcos Luciano Bruschi [1,*]

[1] Postgraduate Program in Pharmaceutical Sciences, Laboratory of Research and Development of Drug Delivery Systems, Department of Pharmacy, State University of Maringa, Maringa, 87020-900, PR, Brazil; rafaelasaids@gmail.com (R.S.d.S.); jessicabassidasilva@gmail.com (J.B.d.S.); helenrosseto@gmail.com (H.C.R.); camilaf.vecchi@gmail.com (C.F.V.)

[2] Postgraduate Program in Chemistry, Department of Chemistry, Research Nucleus in Photodynamic Systems, State University of Maringa, Maringa, 87020-900, PR, Brazil; katieli_souza@hotmail.com (K.d.S.S.C.); wcaetano@uem.br (W.C.)

* Correspondence: mlbruschi@uem.br; Tel.: +55-44-3011-4870

Table S1. Mean globule size and polydispersity index (PI) of dispersed phase of the emulgels containing PF or AN oils just after the preparation (T0) and after 12 days (T12) of the ice-thawing cycle.

Formulations	Mean size (µm)		PI	
	T_0	T_{12}	T_0	T_{12}
F1	32.13 ± 13.29	29.75 ± 11.89	0.6	0.6
F2	25.03 ± 13.01	27.63 ± 10.03	0.5	0.5
F3	27.11 ± 8.36	33.37 ± 12.66	0.4	0.5
F7	34.04 ± 11.25	37.67 ± 19.05	0.5	0.6
F8	27.28 ± 9.02	28.86 ± 9.77	0.5	0.5
F9	26.94 ± 10.71	25.13 ± 6.74	0.6	0.4
F10	43.49 ± 16.83	57.45 ± 40.99	0.6	1.2
F11	37.16 ± 16.10	46.92 ± 38.24	0.7	0.9
F12	36.45 ± 13.73	46.84 ± 48.19	0.6	1.0
F16	39.02 ± 15.75	54.34 ± 31.86	0.7	0.9
F17	38.20 ± 16.08	52.46 ± 36.71	0.7	0.4
F18	40.83 ± 16.80	47.78 ± 29.01	0.7	0.8
F19	39.84 ± 14.51	27.91 ± 9.99	0.6	0.4
F20	30.93 ± 10.73	32.40 ± 12.29	0.5	0.5
F21	32.35 ± 11.18	24.62 ± 8.40	0.5	0.4
F25	34.24 ± 10.65	34.99 ± 12.30	0.5	0.5
F26	28.39 ± 8.00	31.10 ± 8.95	0.4	0.4
F27	29.90 ± 8.91	30.68 ± 9.81	0.4	0.5

*± Standard deviation.

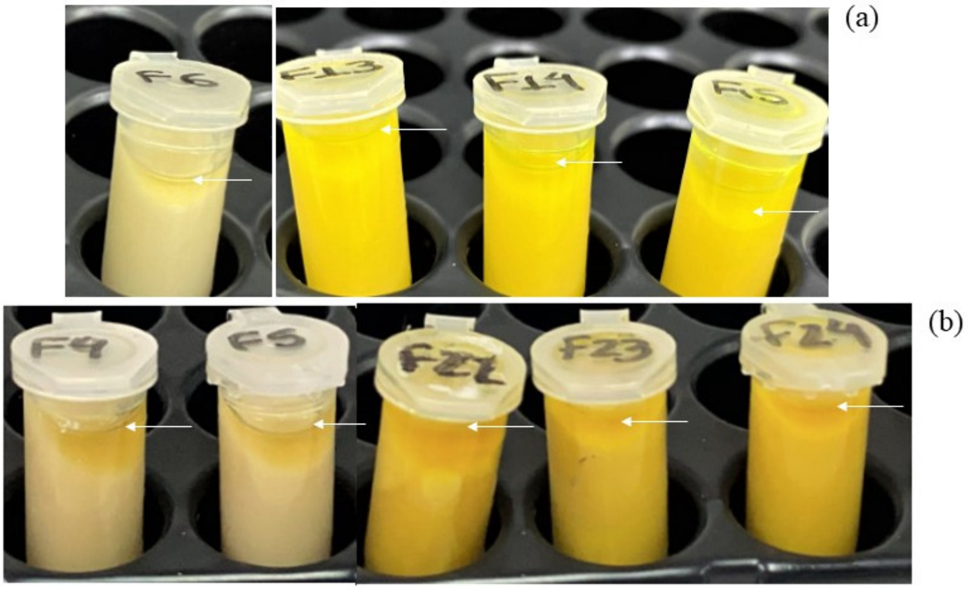

Figure S1. Phase separation of the systems during the physicochemical stability analysis at time zero (a): F6, F13, F14, F15; and after 12 days (b): F4, F5, F22, F23, and F24.

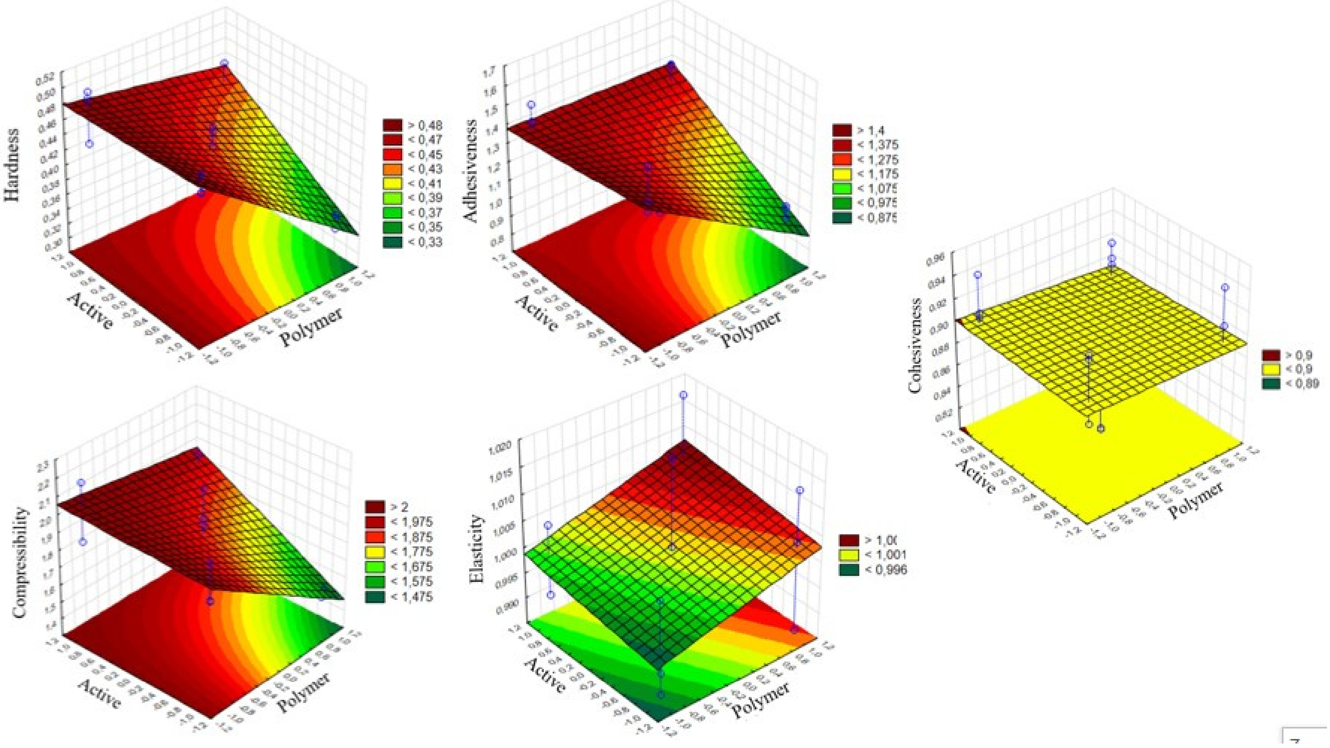

Figure S2. Response surface plots of mechanical properties (hardness, compressibility, adhesiveness, elasticity and cohesiveness) of PF emulgels influenced by different polymers (C934P, C974P, PC), different type of bioactive (PE, CUR, PE+CUR) at 25 °C. The color scale is indicated in each figure and shows the isoparametric values.

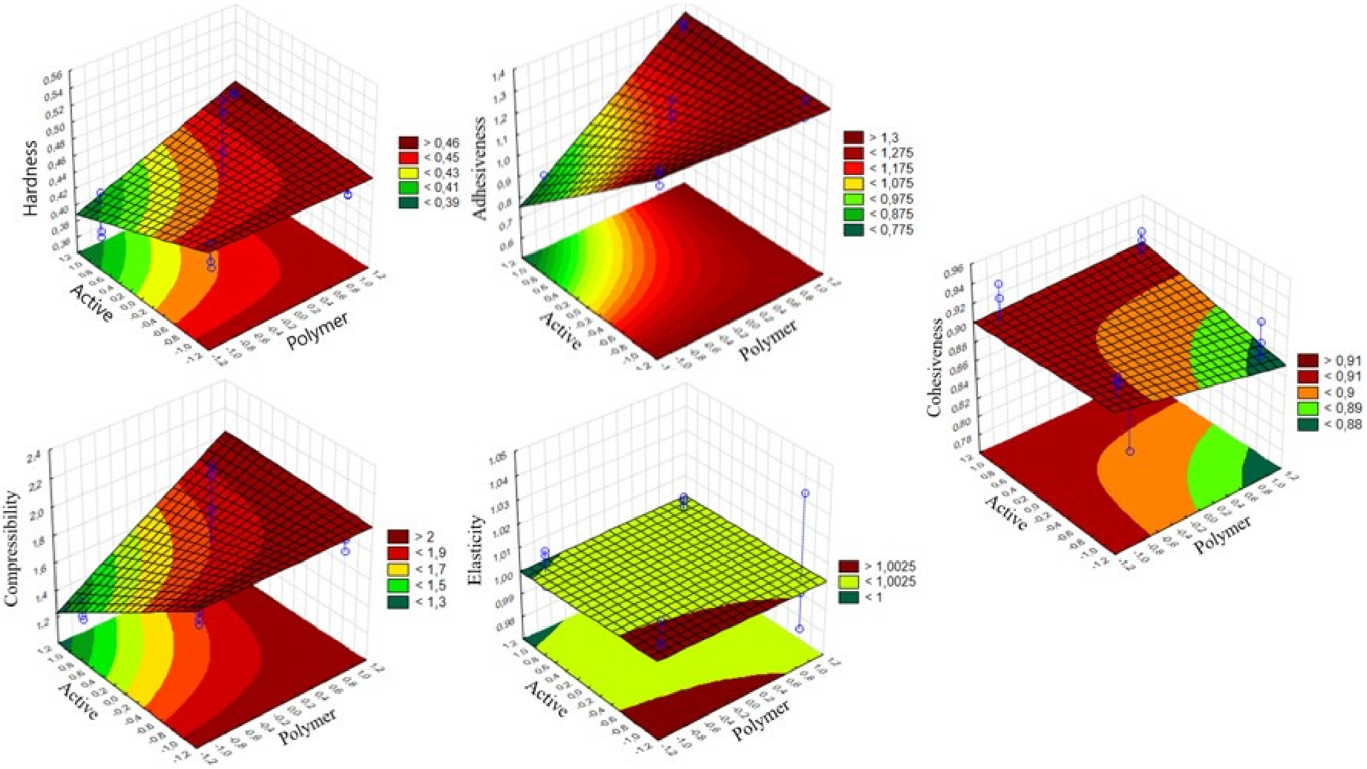

Figure S3. Response surface plots of mechanical properties (hardness, compressibility, adhesiveness, elasticity and cohesiveness) of PF emulgels influenced by different polymers (C934P, C974P, PC), different type of bioactive (PE, CUR, PE+CUR) at 34 °C. The color scale is indicated in each figure and shows the isoparametric values.

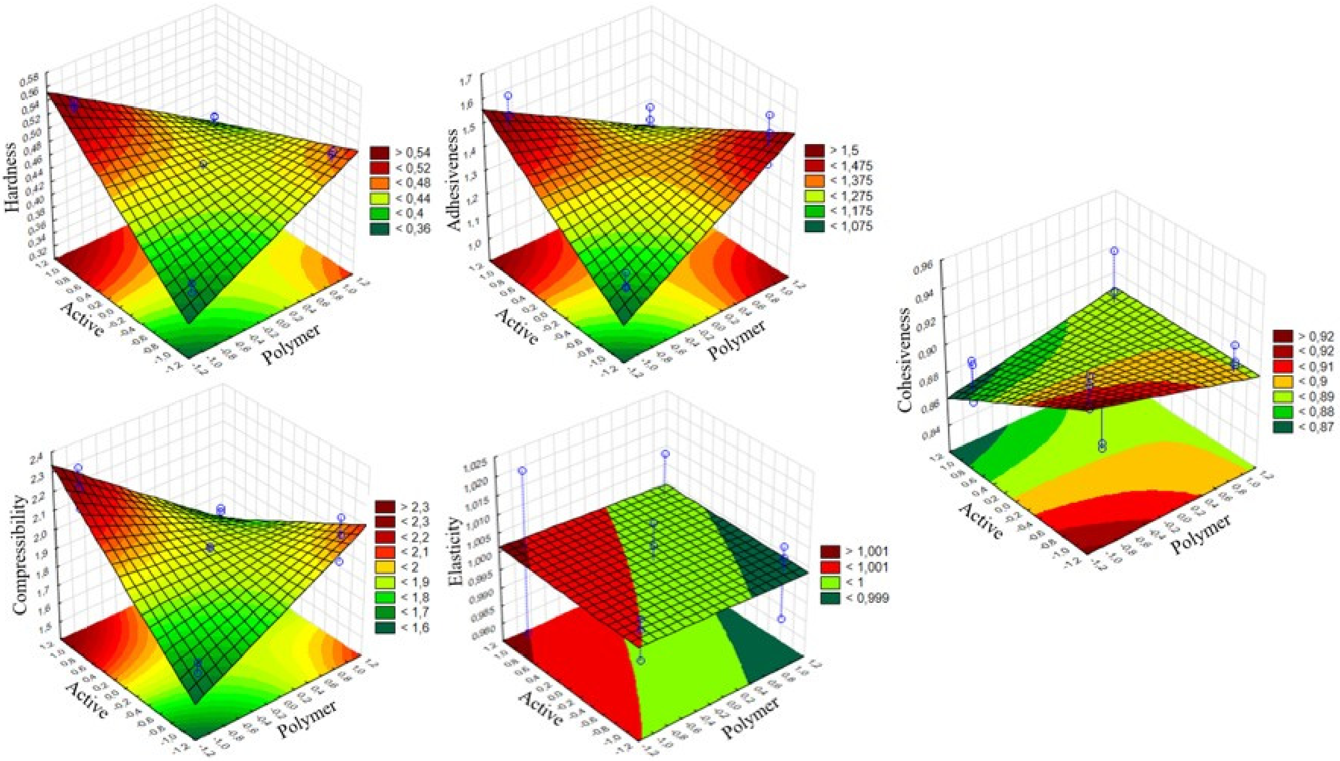

Figure S4. Response surface plots of mechanical properties (hardness, compressibility, adhesiveness, elasticity and cohesiveness) of AN emulgels influenced by different polymers (C934P, C974P, PC), different type of bioactive (PE, CUR, PE+CUR) at 25 °C. The color scale is indicated in each figure and shows the isoparametric values.

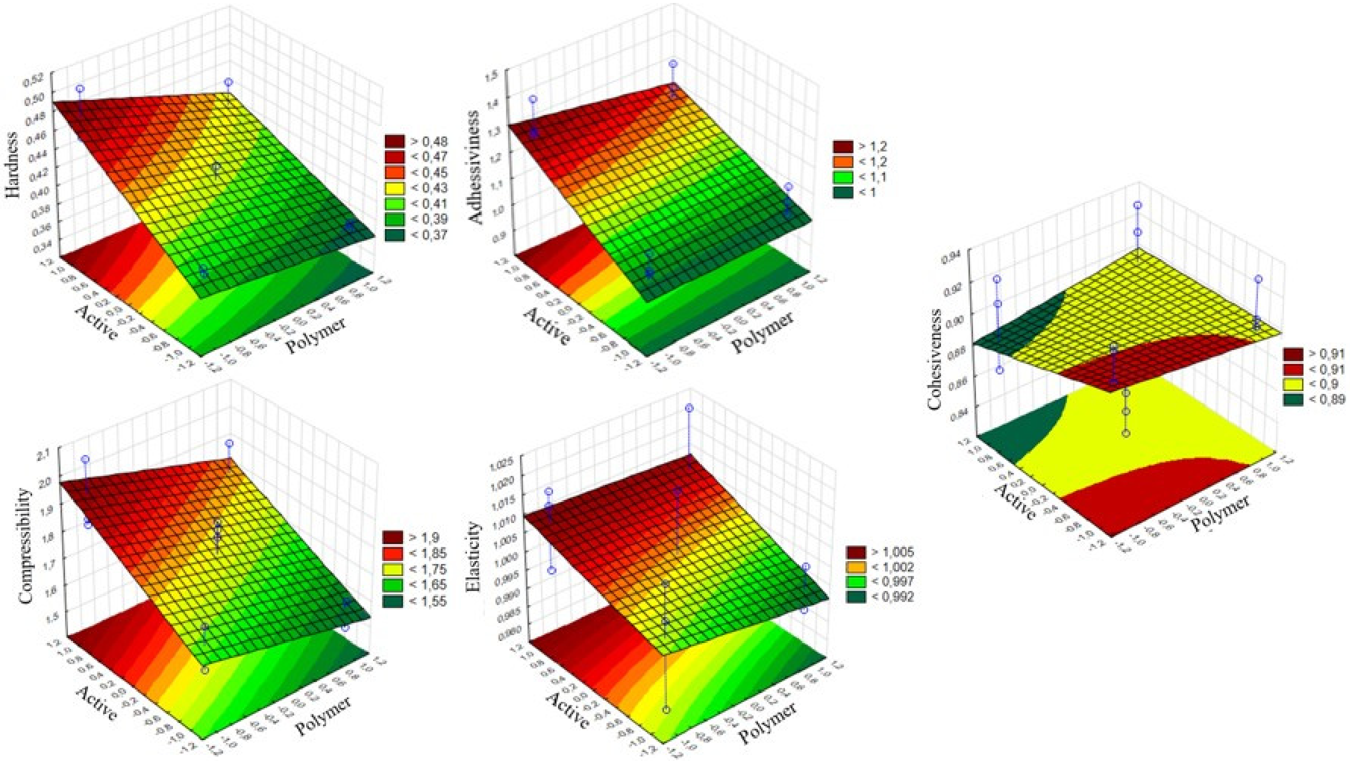

Figure S5. Response surface plots of mechanical properties (hardness, compressibility, adhesiveness, elasticity and cohesiveness) of AN emulgels influenced by different polymers (C934P, C974P, PC), different type of bioactive (PE, CUR, PE+CUR) at 34 °C. The color scale is indicated in each figure and shows the isoparametric values.

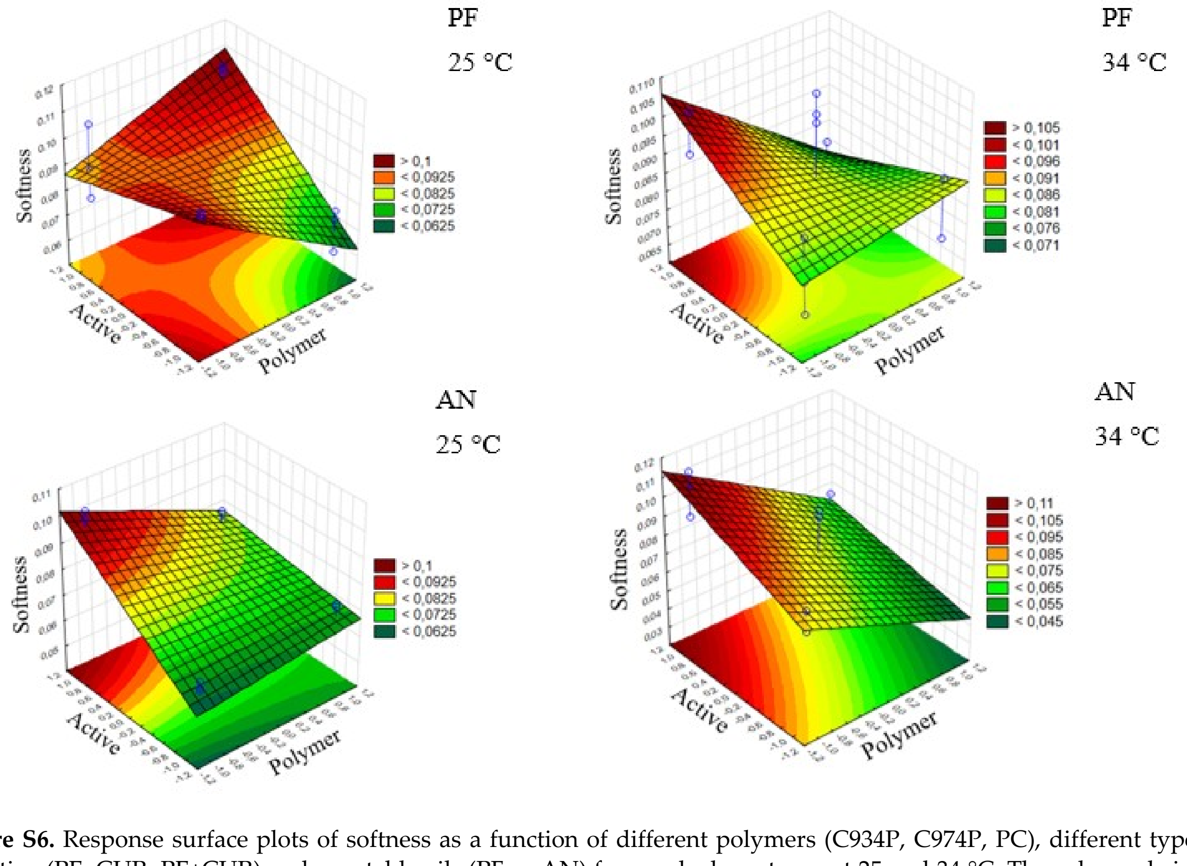

Figure S6. Response surface plots of softness as a function of different polymers (C934P, C974P, PC), different type of bioactive (PE, CUR, PE+CUR) and vegetable oils (PF or AN) for emulgels systems, at 25 and 34 °C. The color scale is indicated in each figure and shows the isoparametric values.

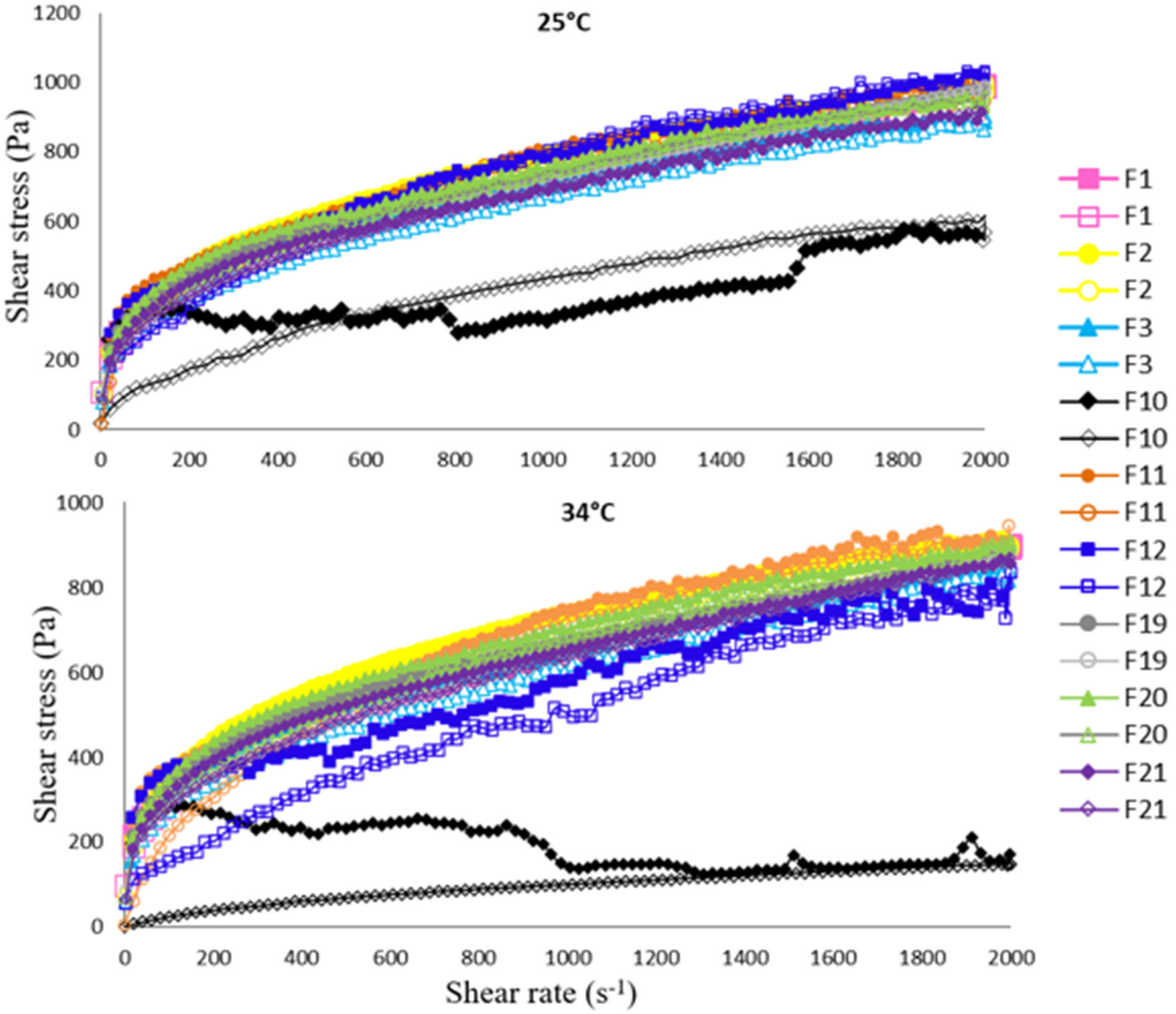

Figure S7. Flow curves of the formulations containing PF at different temperatures. Closed symbol represents the up-curve and open symbol represents the downcurve. Standard deviations have been omitted for clarity; however, in all cases, the relative standard deviation of replicate analysis was less than 10%.

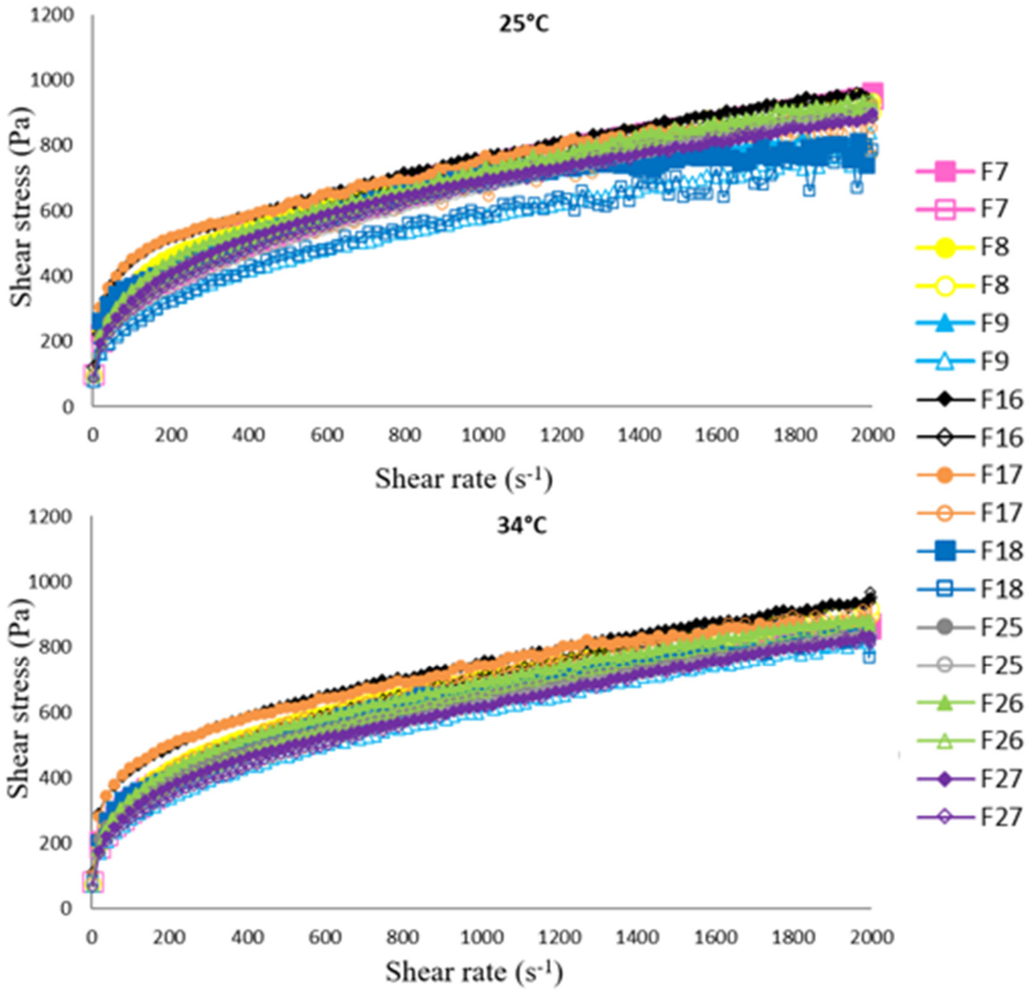

Figure S8. Flow curves of the formulations containing AN at different temperatures. Closed symbol represents the upcurve and open symbol represents the downcurve. Standard deviations have been omitted for clarity; however, in all cases, the relative standard deviation of replicate analysis was less than 10%.

Table S2. Relative standard deviation (%) of emulgels systems F1, F2 and F3, containing PF and PE: elastic modulus (G') and viscous modulus (G") at 25 and 34 °C.

	Relative standard deviation (%)											
	F1				F2				F3			
	25 °C		34 °C		25 °C		34 °C		25 °C		34 °C	
Frequency (Hz)	G′	G″	G′	G″	G′	G″	G′	G″	G′	G″	G′	G″
0.10	6.84	6.93	6.10	5.57	8.72	9.37	4.32	7.44	1.44	6.94	4.79	3.82
0.15	5.73	7.55	1.93	3.02	5.00	3.74	1.19	5.67	2.12	2.33	1.88	9.37
0.22	5.14	6.43	1.58	2.79	4.09	7.12	1.66	9.14	2.91	4.47	3.89	2.93
0.32	1.73	8.24	1.92	7.75	3.88	9.07	1.93	4.05	3.45	6.02	3.73	8.39
0.46	2.39	9.97	2.07	1.07	4.16	8.20	2.24	1.57	3.74	8.31	0.49	7.15
0.68	2.13	7.87	2.04	7.02	4.86	6.87	2.33	3.03	3.87	8.44	3.28	4.11
1.00	1.70	5.71	2.13	5.86	5.03	8.84	2.41	1.72	3.78	7.93	0.60	8.90
1.47	1.91	8.39	2.19	4.71	4.93	3.99	2.31	2.55	3.79	7.37	7.74	3.57
2.15	1.24	3.26	2.19	4.42	5.69	3.95	2.20	1.10	3.70	6.74	9.74	8.71
3.16	2.05	4.05	2.07	2.20	4.89	3.03	2.16	0.17	3.58	5.77	8.64	8.40
4.64	1.37	4.27	1.99	2.35	4.90	4.84	2.17	1.29	3.46	4.59	6.16	9.97
6.81	0.56	3.80	1.86	2.03	4.46	1.10	1.93	2.18	3.14	4.15	5.37	3.12
10.00	1.53	5.02	1.90	0.86	4.50	6.24	1.44	1.59	2.59	2.55	8.58	5.35

Table S3. Relative standard deviation (%) of emulgels systems F7, F8 and F9, containing AN and PE: elastic modulus (G') and viscous modulus (G") at 25 and 34 °C.

	Relative standard deviation (%)											
	**F7**				**F8**				**F9**			
	25 °C		34 °C		25 °C		34 °C		25 °C		34 °C	
Frequency (Hz)	G′	G″	G′	G″	G′	G″	G′	G″	G′	G″	G′	G″
0.10	0.75	7.45	4.01	1.94	6.12	5.79	3.36	5.23	8.63	5.46	7.84	3.78
0.15	1.38	1.05	6.75	5.25	1.71	3.62	4.33	3.97	6.39	6.53	7.71	3.50
0.22	1.86	8.86	9.82	4.40	2.48	3.28	3.87	1.97	8.38	7.52	7.82	7.09
0.32	2.20	3.15	6.28	8.75	6.18	6.94	3.79	2.54	2.34	9.76	7.88	5.42
0.46	2.31	5.33	6.02	1.82	7.95	5.44	3.89	0.80	3.42	4.42	7.96	4.45
0.68	2.49	9.54	2.08	6.09	5.68	4.84	3.87	6.54	4.31	4.15	8.00	0.72
1.00	2.63	8.88	4.37	8.01	4.37	9.91	3.76	3.01	3.77	8.81	7.82	0.17
1.47	2.70	7.83	6.71	8.03	4.98	9.84	3.73	9.81	5.01	0.95	7.70	1.01
2.15	2.79	7.17	6.71	6.59	4.00	8.19	3.89	8.60	2.15	9.57	7.58	0.87
3.16	2.79	6.34	6.67	8.32	6.59	2.46	3.87	8.76	5.10	3.84	7.44	2.36
4.64	2.74	4.89	6.29	7.04	3.36	6.00	3.90	6.51	5.26	3.21	7.17	2.86
6.81	2.63	4.61	5.92	7.31	4.97	3.38	3.88	8.01	3.89	1.18	6.86	3.06
10.00	2.17	3.33	4.94	2.43	1.51	8.67	3.36	2.40	0.84	8.86	6.27	6.23

Table S4. Relative standard deviation (%) of emulgels systems F10, F11 and F12, containing PF and CUR: elastic modulus (G') and viscous modulus (G") at 25 and 34 °C.

	Relative standard deviation (%)											
	**F10**				**F11**				**F12**			
	25 °C		34 °C		25 °C		34 °C		25 °C		34 °C	
Frequency (Hz)	G′	G″	G′	G″	G′	G″	G′	G″	G′	G″	G′	G″
0.10	0.85	1.17	8.40	5.99	1.35	2.75	6.04	6.30	1.68	5.75	8.46	2.43
0.15	1.89	9.18	5.75	4.97	1.39	2.70	0.09	6.33	0.48	0.33	9.73	8.22
0.22	4.91	5.70	7.98	7.37	1.36	2.67	4.84	5.67	1.11	1.89	9.23	8.42
0.32	9.48	5.54	4.99	4.95	1.36	2.48	0.00	6.07	0.93	3.98	9.41	6.01
0.46	8.72	5.56	3.08	3.34	1.38	2.44	0.72	5.23	0.16	9.79	9.55	7.90
0.68	8.00	4.94	1.74	4.76	1.47	2.42	1.21	0.99	0.74	9.03	9.59	6.56
1.00	7.40	4.20	0.65	5.68	1.45	2.08	1.48	9.27	0.77	3.55	9.59	6.16
1.47	6.79	8.98	1.94	2.64	0.45	2.30	1.52	9.24	0.15	4.77	9.64	7.87
2.15	6.29	7.95	4.30	4.29	1.46	2.23	2.17	1.05	1.23	4.81	9.78	8.62
3.16	5.97	7.39	5.42	2.99	1.50	2.40	2.12	9.68	0.66	9.14	9.34	6.25
4.64	5.34	5.80	6.00	8.91	1.47	2.44	1.74	2.53	0.22	9.20	9.26	7.13
6.81	5.18	7.55	7.51	6.10	1.36	3.71	1.79	0.43	0.14	1.48	9.86	7.80
10.00	4.87	1.47	7.47	2.62	1.25	2.09	1.94	7.64	8.29	9.26	8.39	9.94

Table S5. Relative standard deviation (%) of emulgels systems F16, F17 and F18, containing AN and CUR: elastic modulus (G') and viscous modulus (G") at 25 and 34 °C.

**Relative standard deviation (%)**												
	**F16**				**F17**				**F18**			
	25 °C		34 °C		25 °C		34 °C		25 °C		34 °C	
Frequency (Hz)	G′	G″	G′	G″	G′	G″	G′	G″	G′	G″	G′	G″
0.10	8.62	9.71	4.49	9.88	2.42	8.82	5.22	9.78	8.23	8.78	2.83	7.22
0.15	6.11	8.76	2.36	9.92	9.43	7.14	4.71	9.96	9.82	8.70	3.21	4.31
0.22	5.55	9.34	2.41	6.58	5.98	9.02	4.65	9.00	8.71	8.30	3.23	5.66
0.32	2.51	8.63	2.85	3.96	2.39	9.59	4.74	7.44	0.36	8.22	3.17	4.21
0.46	2.29	3.82	2.64	2.57	2.39	6.94	4.84	7.04	6.10	9.21	3.06	2.89
0.68	3.99	4.86	2.58	2.75	2.99	9.16	4.90	6.45	4.82	8.98	2.98	4.30
1.00	4.85	1.55	2.63	2.34	2.39	4.46	4.81	5.98	0.26	4.05	2.95	3.99
1.47	2.94	2.53	2.61	1.74	3.87	1.66	4.84	5.06	2.23	7.73	2.90	3.58
2.15	1.49	0.98	2.57	3.43	2.78	8.17	4.89	6.26	2.05	9.04	2.80	3.52
3.16	3.10	3.18	2.95	2.76	3.71	8.07	4.80	5.43	1.15	9.64	2.91	2.29
4.64	2.99	5.52	2.64	3.05	3.99	4.03	4.80	4.51	1.70	6.59	2.74	2.56
6.81	3.39	5.19	2.68	1.70	3.71	4.37	4.62	6.07	1.57	9.38	2.69	2.85
10.00	1.57	4.44	2.49	1.53	4.49	9.57	4.14	8.00	4.48	9.02	2.37	6.29

Table S6. Relative standard deviation (%) of emulgels systems F19, F20 and F21, containing PF and PE + CUR: elastic modulus (G') and viscous modulus (G") at 25 and 34 °C.

Relative standard deviation (%)												
	F19				F20				F21			
	25 °C		34 °C		25 °C		34 °C		25 °C		34 °C	
Frequency (Hz)	G′	G″	G′	G″	G′	G″	G′	G″	G′	G″	G′	G″
0.10	1.02	0.01	7.13	9.93	9.66	9.34	2.10	9.36	9.16	9.48	1.90	9.79
0.15	7.53	0.02	3.86	3.91	4.39	5.38	3.51	9.13	9.63	9.12	2.12	9.51
0.22	9.64	0.03	3.55	0.67	7.87	7.59	3.83	6.34	2.53	9.50	3.46	8.93
0.32	8.46	0.05	3.34	2.09	7.10	9.67	3.72	6.81	3.29	9.86	4.18	8.12
0.46	9.55	0.01	3.33	2.73	9.80	5.32	3.59	6.86	5.41	9.88	4.02	8.55
0.68	9.65	0.05	3.26	1.85	6.31	9.45	3.73	6.31	5.52	9.31	3.87	8.18
1.00	9.26	0.01	3.23	2.02	5.99	9.63	3.61	4.46	4.31	8.70	3.41	9.58
1.47	7.17	0.03	3.23	1.74	4.14	9.88	3.59	2.51	4.07	8.46	3.23	7.79
2.15	5.82	0.04	3.22	1.98	4.15	5.65	3.61	9.63	3.81	8.42	2.86	5.28
3.16	5.38	0.01	3.15	2.18	5.29	4.89	3.53	8.28	2.89	6.91	2.51	4.30
4.64	5.82	0.02	3.08	1.14	4.89	8.84	3.50	5.52	4.19	9.94	2.38	2.08
6.81	3.40	0.06	2.98	1.56	3.09	2.61	3.49	4.02	5.18	9.82	2.08	0.56
10.00	4.68	0.19	2.82	3.71	5.30	3.93	3.35	5.49	2.76	9.00	1.98	2.48

Table S7. Relative standard deviation (%) of emulgels systems F25, F26 and F27, containing PF and PE + CUR: elastic modulus (G') and viscous modulus (G") at 25 and 34 °C.

Relative standard deviation (%)												
	**F25**				**F26**				**F27**			
	25 °C		34 °C		25 °C		34 °C		25 °C		34 °C	
Frequency (Hz)	G′	G″	G′	G″	G′	G″	G′	G″	G′	G″	G′	G″
0.10	8.18	5.62	5.05	9.64	9.44	6.06	7.17	8.68	9.46	9.83	5.16	3.43
0.15	9.39	9.31	5.10	8.39	9.97	7.64	7.96	8.80	7.69	9.73	5.30	8.89
0.22	4.89	9.75	5.34	6.44	4.04	9.11	6.44	9.63	8.50	4.77	5.37	3.35
0.32	0.67	8.57	5.52	5.52	1.84	9.00	5.42	6.22	4.68	5.19	4.26	6.11
0.46	3.97	5.24	5.74	4.14	1.37	8.13	4.84	1.43	6.62	2.46	4.89	7.12
0.68	0.13	6.78	5.78	5.15	2.33	8.37	4.11	6.46	3.03	4.89	4.43	8.40
1.00	1.31	3.31	5.76	4.86	2.35	8.41	3.89	6.28	1.98	9.91	4.13	7.54
1.47	1.14	1.59	5.77	5.37	1.89	7.64	3.49	0.87	3.57	9.55	3.93	6.23
2.15	2.39	8.59	5.72	5.35	2.76	6.06	3.38	1.76	2.24	8.74	3.67	5.46
3.16	1.34	9.47	5.61	5.90	1.64	4.37	3.36	2.45	2.60	3.19	3.43	4.15
4.64	1.55	7.75	5.42	4.97	2.29	4.90	3.15	2.59	9.58	8.03	3.19	4.06
6.81	1.52	4.30	4.90	5.67	2.08	9.29	2.96	3.06	2.83	7.17	2.95	5.19
10.00	1.50	3.64	4.37	5.61	2.80	9.68	2.57	4.68	2.51	4.76	2.37	3.80

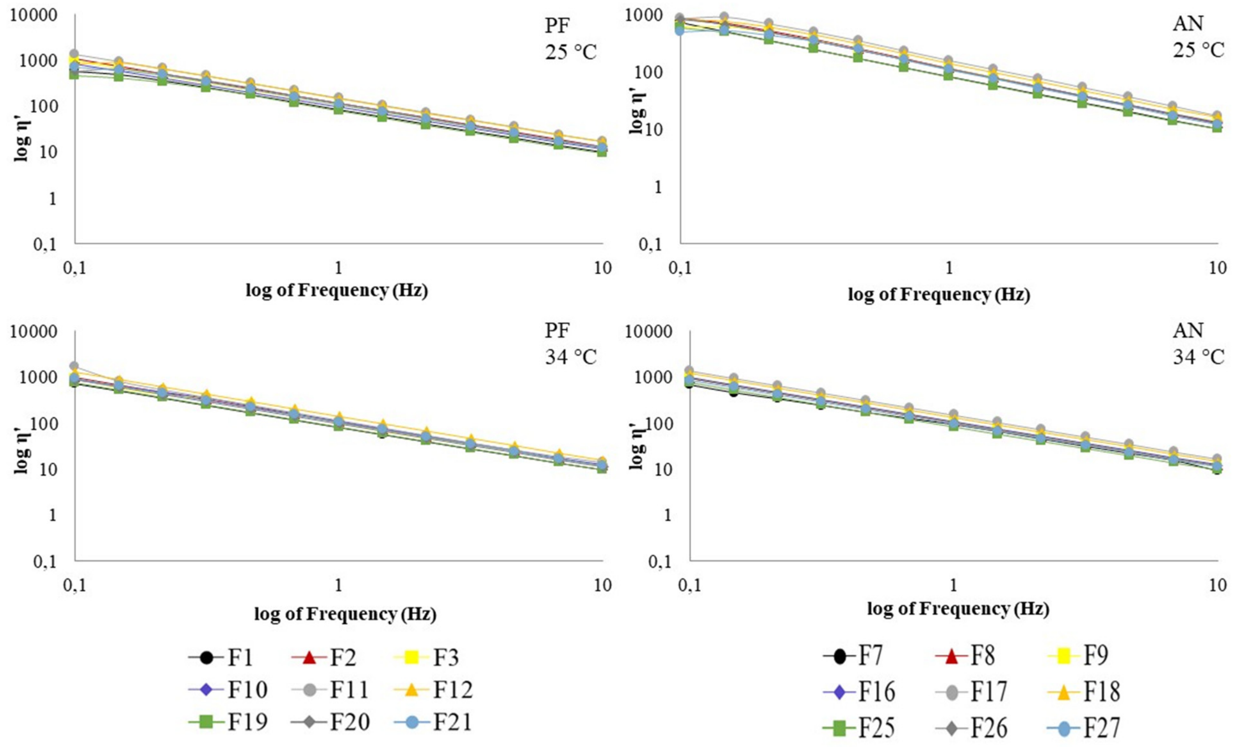

Figure S9. Viscosity ή as a function of frequency of formulations without PF and AN oil at temperatures 25 and 34 °C. Each point is the mean of at least three replicates. Standard deviations have been omitted for clarity; however, in all cases, the relative standard deviation of replicate analysis was less than 10%.

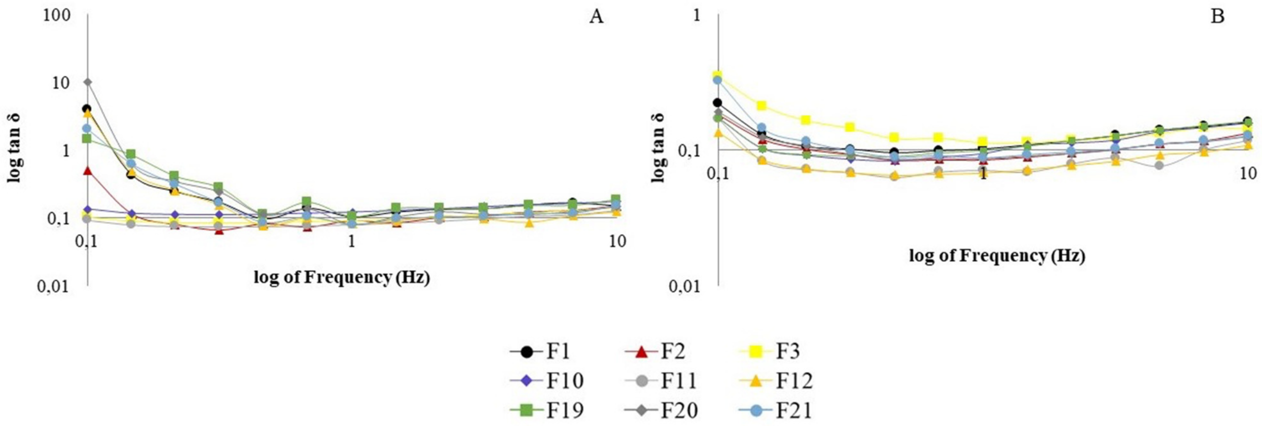

Figure S10. Tan δ as a function of frequency of formulations containing PF at temperatures of 25 (A) and 34 °C (B). Each point is the mean of at least three replicates. Standard deviations have been omitted for clarity; however, in all cases, the relative standard deviation of replicate analysis was less than 10%.

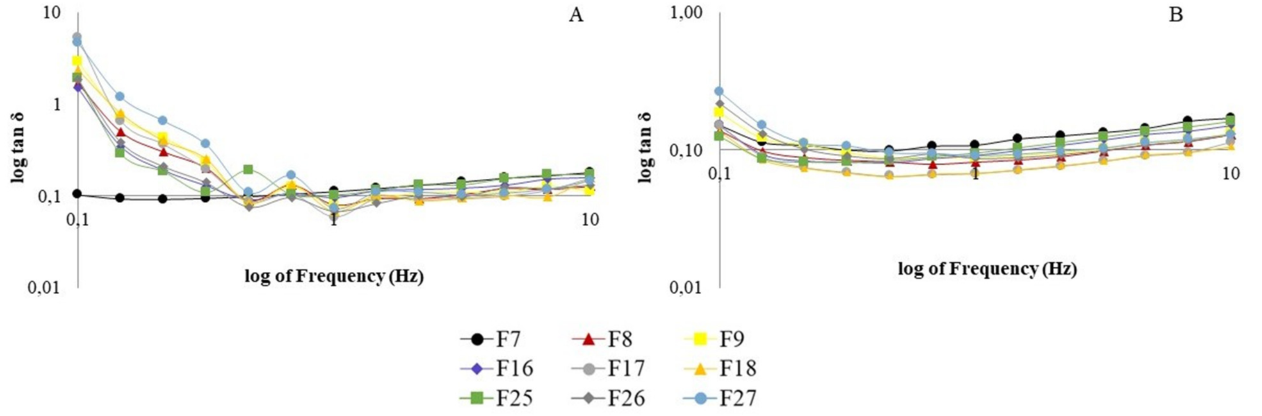

Figure S11. Tan δ as a function of frequency of formulations containing AN at temperatures of 25 (A) and 34 °C (B). Each point is the mean of at least three replicates. Standard deviations have been omitted for clarity; however, in all cases, the relative standard deviation of replicate analysis was less than 10%.
